# Remediation of Crude Oil-Polluted Soil by the Bacterial Rhizosphere Community of *Suaeda Salsa* Revealed by 16S rRNA Genes

**DOI:** 10.3390/ijerph17051471

**Published:** 2020-02-25

**Authors:** Yilei Yu, Yinghua Zhang, Nana Zhao, Jia Guo, Weigang Xu, Muyuan Ma, Xiaoxia Li

**Affiliations:** 1 Institute of Wetland Research, Chinese Academy of Forestry, Beijing 100091, China; yuyilei1222@126.com (Y.Y.); gjiaty@163.com (J.G.); xuweigang@foxmail.com (W.X.); xiaogu67@sina.com (M.M.); kindxiaoxia@163.com (X.L.); 2 Beijing Key Laboratory of Wetland Services and Restoration, Beijing 100091, China; 3 Key Laboratory of Water Cycle and Related Land Surface Processes, Institute of Geographic Sciences and Natural Resources Research, Chinese Academy of Sciences, Beijing 100101, China; zhangyinghua@igsnrr.ac.cn

**Keywords:** crude oil pollution, bacterial community, soil rhizosphere

## Abstract

Crude oil pollution of soil is a serious environmental issue, and bioremediation using plants and microorganisms is a natural and sustainable method for its restoration. Pot incubation of a two-factor randomized block (plants with two levels, and crude oil with three levels) was designed to investigate the rhizosphere bacterial community of *Suaeda salsa* (L.) Pall. Crude oil contamination of soil was studied at different levels: 2 g/kg (low), 4 g/kg (medium), and 6 g/kg (high) levels. In this study, the physicochemical properties of the collected rhizosphere soil were analyzed. Moreover, the soil bacteria were further identified using the 16S rRNA gene. The effects of *S. salsa* and crude oil and their interaction on the physiochemical properties of the soil and crude oil degradation were found to be significant. Crude oil significantly influenced the diversity and evenness of bacteria, while the effects of *S. salsa* and interaction with crude oil were not significant. *Proteobacteria* were found to be dominant at the phylum level. Meanwhile, at the genera level, *Saccharibacteria* and *Alcanivorax* increased significantly in the low and medium contamination treatment groups with *S. salsa*, whereas *Saccharibacteria* and *Desulfuromonas* were prevalent in the high contamination treatment group. High crude oil contamination led to a significant decrease in the bacterial diversity in soil, while the effects of *S. salsa* and its interaction were not significant. Despite the highest abundance of crude oil degradation bacteria, *S. salsa* reduced crude oil degradation bacteria and increased bacteria related to sulfur, phosphorus, and nitrogen cycling in the low and high contamination group, whereas the opposite effect was observed for the medium contamination treatment group. The abundance of most crude oil degradation bacteria is negatively correlated with crude oil content. Nitrogen cycling bacteria are sensitive to the total nitrogen, total phosphorus, ammonia nitrogen, and nitrate nitrogen, and pH of the soil. Sulfur cycling bacteria are sensitive to aromatic hydrocarbons, saturated hydrocarbons, and asphaltene in soil. This research is helpful for further studying the mechanism of synergistic degradation by *S. salsa* and bacteria.

## 1. Introduction

Environmental pollution with crude oil has been a serious worldwide environmental concern [1,2]. Crude oil causes drinking water pollution [3,4], decline in the water and air quality as well as soil fertility, and wastage of non-renewable resources [5,6,7,8,9]. This damage to the ecosystem is due to the accidental leakage of crude oil or its derivatives. Petroleum hydrocarbons could reduce the aeration and water permeability of soil by filling the soil pores, thereby causing ecological and toxicological effects on plants and the destruction of the natural state of the oilfield [10]. Moreover, carcinogenic and mutagenic crude oil compounds could cause fatal mutations in genetic material even at low levels of pollution due to their persistence and biological toxicity [11,12,13,14]. Therefore, they have been the subject of focus, especially because of their structural complexity and hydrophobicity [14,15,16]. Crude oil is mainly composed of carbon and hydrogen, but also contains nitrogen, oxygen, sulfur, and various metals [17,18]. It consists of four main components: (1) saturated hydrocarbon, (2) aromatic hydrocarbon, (3) asphaltene, and (4) colloid [19,20].

The cost of many conventional physical and chemical remediation methods (soil cleaning, chemical reduction or oxidation of contaminants, and incineration) are expensive due to the use of ex situ treatments. In addition, they also usually cause secondary pollution problems as a result of the transport of contaminants and chemical reagents [21,22]. Bioremediation of terrestrial and aquatic ecosystems polluted with crude oil has been realized using green plants, microbes, or their metabolic activity [23]. The advantages of bioremediation methods include minimal on-site operational costs, no secondary pollution, and greater public acceptance [24,25]. Biological methods include: (1) degradation of carbon-based chemicals using microorganisms (fungi/bacteria), (2) use of plants, especially fast-growing plants with large biomass, and (3) storage or stabilization by soil animals, and (4) combined use of plants and bacteria, referred to as microbe-assisted phytoremediation [16,26,27]. Previously, plants have been actually applied in the remediation of crude oil contamination [28]. However, the biodegradation rate is at the lowest level when the crude oil contamination of soil is at 40 g/kg [29]. Dynamic synergy between plant roots and soil microbes has received considerable attention owing to the possible role of bacteria in plant growth and the degradation of petroleum hydrocarbons [30]. Numerous microorganisms such as bacteria and fungi inhabit the rhizosphere, and contribute to plant restoration in the root zone [9,31]. For example, the root activity of alfalfa and perennial ryegrass increases the amount of rhizosphere bacteria capable of degrading crude oil in the soil [32]. The successful application of rhizosphere restoration depends to a large extent on the survival and establishment of rhizobacteria. However, the complete mechanism of this process is still not known [33].

The Yellow River Delta is one of the most representative coastal wetlands in China. Further, the second-largest oil field (Shengli Oilfield) in China is also located at the Yellow River Delta. Crude oil pollution has become an important factor that degrades wetland ecosystems [34]. Studies have demonstrated that the background value of petroleum pollution was 67.22 mg/kg in the Yellow River Delta in the period of 1980s to 1990s, and the average content of crude oil in soil was 235.56 mg/kg [35,36]. In 2001, the content of petroleum hydrocarbons close to Gudong and Shengtuo oilfields was greater than 500 mg/kg, and the highest petroleum content in the soil of Gudong Oilfield was 700 mg/kg [37]. Meanwhile, since 2006, it has been found that most of the soil crude oil content in the 100 m^2^ area around oil wells is higher than 500 mg/kg. Moreover, the crude oil content in the oil spill area is as high as 6320 mg/kg. In addition, the polluted soil area in the oil-spill surroundings accounts for 24% of the total area [38,39].

Halophyte community that consists of herbaceous plants is the principal type of vegetation in the Yellow River delta. Among these plants, *Suaeda salsa* (L.) Pall is a dominant species with strong salt tolerance that is widely distributed [40,41]. Considering a high crude oil contamination of 6 g/kg, we planned to use *S. salsa* to remediate the soil polluted with crude oil, and to investigate the rhizosphere bacterial community of *S. salsa* cultivated in the contaminated soil. Hence, we designed the experiment with the following objectives: (1) to investigate the physicochemical characteristics of soils contaminated by crude oil; (2) characterize the feature of bacterial community in the rhizosphere of *S. salsa*; (3) understand the relationship between the bacterial community and soil characteristics. This study would be useful for the bioremediation of soil polluted with crude oil.

## 2. Materials and Methods

### 2.1. Experimental Designs

In order to study the characteristics of rhizosphere bacterial community of *S. salsa*, pot incubation of two-factor randomized block (plants with two levels and crude oil with three levels) was designed. Detailed information on the pot experiments is provided in the following. Considering the actual crude oil contamination reported in the Yellow River delta, three crude oil pollution levels were set in the experiments, which are 2 g/kg (C1), 4 g/kg (C2), and 6 g/kg (C3), respectively. Oil-contaminated soil with no plant (*S. salsa*) was used as the control to observe the decay characteristics in the absence of the plant. And P0 and P2 means no plant and with plant (*S. salsa*), respectively. Each control and treatment experiment was repeated thrice.

The annual average temperature, humidity, and precipitation of the study site are 13.9 °C 63%, and 554 mm, respectively. Further, the soil type and texture are coastal saline soil and sandy loam, respectively. In May 2015, soil was collected from natural soil at a depth of 0–20 cm in Dongying Halophyte garden (N: 37°24′47.32″; E: 118°39′49.68″; altitude: 1 m) of the Yellow River delta, Shandong Province. The soil was first passed through a 100-mesh sieve (mesh size: 0.150 mm) and air-dried indoors for three days in sunny weather, and then was stored at 4 °C. The soil properties such as pH, total salt content, ammonium nitrogen, total nitrogen, and total organic matter were found to be 7.60, 5.74 g/kg, 1.09 mg/kg, 7.86 mg/kg, and 4.14 g/kg, respectively. Each pot which both the diameter and height of nearly 20 cm was filled with 4 kg of air-dried soil. The crude oil used in the experiment was collected from a natural well in the Gudong Oilfield in Yellow River delta; the concentrations of saturated hydrocarbon, aromatic hydrocarbon, asphaltene, and colloid in this sample were found to be 482 g/kg, 278 g/kg, 12.5 g/kg, and 11.4 g/kg, respectively. The crude oil was dissolved in petroleum ether, and then the petroleum ether was fully mixed with soil and evenly packed into pots. Thereafter, petroleum ether was let fully volatilized for eliminating its effect.

Seeds of *S. salsa* (collected from a field site in autumn) with a weight of 0.7 g were prepared for each treatment. The seeds were disinfected with 1% potassium permanganate solution for 30 min and then rinsed with distilled water. *S. salsa* was cultivated in pots outside of the Dongying Halophyte garden in May 2015. After 10 days of sprouting, the seedlings were thinned out, and 45 seedlings per pot were kept. Quantitative watering was carried out according to different growth periods of *S. salsa* for both the control and treatment group in the same period. Fertilizers and pesticides were not used during the growth period. Rhizosphere soil samples were collected from *S. salsa* pots in September (the growth period), and then *S. salsa* was harvested in November 2015. The dry weights of *S. salsa* from the low (P2C1), medium (P2C2), and high (P2C3) contamination treatment groups in the harvest period are 26.18 g, 4.29 g, and 2.20 g, respectively.

### 2.2. Soil Sampling

The rhizosphere is the surface of root and the surrounding soil layer, without a fixed interface. It is closely related to plant species, plant growth and development stages, root age, and root environment. In our study, considering the root depth, soil within 1 cm of the root from different depths (0–5 cm) was collected using plastic spoons. Disposable plastic spoons were disinfected and sterilized with ultraviolet light. First, the rhizosphere soil was collected and placed in 50 mL sterile tubes, and the tubes containing soils were immediately stored in liquid nitrogen (−80 °C). Then, the samples were kept in a liquid nitrogen jar until their analysis.

Air-dried soil samples (10 g) were placed in 50 mL centrifuge tubes. Then, 30 mL of chloroform was added to each tube and sealed with a lid. The samples were first placed on a shaker for 5 min and subsequently left undisturbed overnight. The next day, hot dip ultrasonic extraction was performed for 1 h in a 55 °C water bath using an ultrasound bath (KQ-600E, Kunshan Company, Kunshan, China). Then, the samples were centrifuged (Sigma 8k, Sigma, Osterode am Harz, Germany) at 4500 r/min for 5 min, and the supernatants were collected by filtration. Hot dip ultrasonic extraction was repeated twice, and the collected samples were dried over anhydrous sodium sulfate, and then dried by cooling. Depending on the oil and gas industry standards [20], chromatographic techniques were used to separate alkanes, aromatic hydrocarbons, and polar substances in the crude oil. The inner diameter and length of the glass chromatography column (C 7 × 400, Beijing Glass Instrument Company, Beijing, China) are 7 mm and 400 mm, respectively. Silica and alumina were used as the solid phase. Silica gel (particle size: 0.177 mm) was extracted with chloroform until there was no fluorescence, and activated in an electrothermal drying oven at 140–150 °C for 8 h; neutral alumina (particle size: 0.149 mm) was activated for 4 h at 400–450 °C. After activation, they were transferred to a desiccator for cooling and placed in a grinding bottle, and then stored in the desiccator. The silica–alumina column was continuously eluted with n-hexane, and the extracted crude oil was passed through a column in which the polar substance was adsorbed by alumina. The column was repeatedly eluted with 30 mL volumes of n-hexane, and then the selected alkane component was collected. After that, the column was eluted with 30 mL of a mixture of dichloromethane and n-hexane (65:35 by volume) to collect the aromatic component. Finally, the column was repeatedly eluted with 30 mL volumes of chloroform and the polar component was collected, which was dried over anhydrous sodium sulfate, and dried by cooling. The total amount of petroleum pollutants and the composition of each family were determined by a weighing method described elsewhere [20]. The pH, ammonium nitrogen (NH_4_-N), nitrate nitrogen (NO_3_-N), total nitrogen (TN), total phosphorus (TP), and soil organic matter (SOM) in the soil were evaluated with reference to *Soil Agrochemical Analysis* (third Edition) [42]. Soil NH_4_–N and NO_3_–N were extracted with 100 mL of a 1 mol L^−1^ KCl solution, shaken in a rotary shaker (140 r min^−1^) for 1 h, and filtered into sampling bottles. Samples were stored frozen until their analysis with a colorimetric continuous flow analyzer (SANT++, Skalar Company, Breda, Netherlands). The SOM was determined by the potassium dichromate titrimetric method using an electric balance (FA-N, Jingqi Company, Shanghai, China). The TN was determined by Kjeldahl digestion using a Kjeldahl analyzer (KjelFlexK-360, Buchi, Flawil, Switzerland). The TP was determined by the molybdenum blue method using a UV-vis spectrophotometer (DB-20R, HALO, Sydney, Australia).

### 2.3. Soil DNA Extraction, Polymerase Chain Reaction (PCR), and Illumina MiSeq Sequencing

Microbial DNA samples from eighteen rhizosphere soil samples of *S. salsa* were extracted in our study using a soil DNA Kit (Omega Bio-tek, Norcross, GA, USA), according to the operation instructions. First, 0.5 g of the soil was added to a 2 mL tube containing 250 mg of beads (Omega Soil DNA Kit 1000, D5625-04). Then, microorganisms in the soil were hit for 5 mini by a grinding instrument at 40 HZ frequency (High-flux tissue machine, WB2017075, Shanghai Wanbai Company, Shanghai, China). Next, impurities were removed by centrifugation, SP2, HTR reagents, etc. Finally, the DNA was adsorbed, washed, and recovered by the Binding Buffer column method. The concentration, purification, and quality of the extracted DNA were evaluated by UV-vis spectrophotometry (NanoDrop 2000 spectrometer, Thermo Scientific, Wilmington, DE, USA) and 1% agarose gel electrophoresis. The V3–V4 region of the bacterial 16S rRNA gene was amplified by PCR (95 °C for 3 min, followed by 27 cycles of heating at 95 °C for 30 s, annealing at 55 °C for 30 s, elongation at 72 °C for 45 s, and final extension at 72 °C for 10 min) using 338F (5′- ACTCCTACGGGAGGCAGCAG-3′) and 806R (5′-GGACTACHVGG GTWTCTAAT-3′) using a thermocycler PCR system (GeneAmp 9700, ABI, Waltham, MA USA). A 20 μL mixture of the PCR amplification system contained 4 μL of 5 × FastPfu buffer, 2 μL of 2.5 mM dNTPs, 0.8 μL of each primer (5 μM), 0.4 μL of FastPfu Polymerase, and 10 ng of the template DNA. The PCR product was recovered using a 2% agarose gel, purified using an AxyPrep DNA gel extraction kit (Axygen Biosciences, Union City, CA, USA), eluted with Tris-HCl, and detected by 2% agarose electrophoresis. Detection and quantification were performed using QuantiFluorTM-ST (Promega, Madison, WI USA).

### 2.4. Statistical Analyses

The purified amplified fragment was constructed into a library of PE 2×300 according to the standard operating protocol of the Illumina MiSeq platform (Illumina, San Diego, CA, USA), and paired-end reading was performed. The original sequences were controlled using Trimmomatic software (Version 3, Majoorbio Company, Shanghai, China) and spliced using FLASH software (version 1.2.7, Majoorbio Company). Raw fastq files were quality-filtered by Trimmomatic and merged by FLASH according to the following criteria: (i) the reads were truncated at any site receiving an average quality score of <20 over a 50 bp sliding window. (ii) Sequences whose overlap being longer than 10 bp were merged according to their overlap with no more than 2 bp mismatch. (iii) Sequences of each sample were separated according to barcodes (exactly matching) and primers (allowing 2 nucleotide mismatching), and reads containing ambiguous bases were removed. The original sequence data had been submitted to NCBI, and the accession number is PRJNA589112.

Bioinformatics data were analyzed on the free online platform of Majorbio I-Sanger Cloud Platform (www.i-sanger.com). R language (https://www.rproject.org/) was used in further analysis. The UPARSE software (version 7.1, http://drive5.com/uparse/) was used to perform OTU clustering on the sequences, according to 97% similarity; chimeras were removed using UCHIME (Version 4.2, http://drive5.com/usearch/manual/uchime_algo.html) software. Cluster analysis was performed using the unweighted pair group method with arithmetic mean (at the OTU level). Each sequence of species was classified by the Bayesian algorithm method using the RDP classifier (http://rdp.cme.msu.edu/) according to 97% similarity levels, comparing the Silva Database (SSU123), and using the alignment threshold of 70%. Relative abundances at phylum and genus level were calculated from the ratio of sequence numbers of species with total numbers.

Good’s coverage (C) represents the ratio of all non-singletons in the total samples. It could be used to discover whether the sequencing data was saturated. Exponential Shannon-Wiener index (e^H′^) and Simpson’s reciprocal index (D_r_) were used to estimate the level of community diversity [43]. Evenness index of species uniformity (J) indicated the distribution of the number of individuals in a community, which is a measure of the proximity of different species in terms of quantity [43]. These parameters were performed by Mothur software (Version 1.39.5, https://www.mothur.org/) on Majorbio I-Sanger Cloud Platform, and the formulas are as follows. During calculation, data quantity will be filtered according to minimum sample sequence numbers (min: 20 219; max: 39465), and this can avoid the difference caused by the different amount of data produced by different samples in the process of analysis:

(1) $$C=1-(\frac{F_{1}}{N})$$

where, *F*_1_ represents the number of singletons and N represents the total number of all OTUs in the sample:

(2) $$H^{\prime }=-\sum_{i=1}^{s}P_{i}\times \ln P_{i},P_{i}=\frac{n_{i}}{N}$$

(3) $$D_{r}=\frac{1}{1-\sum_{i=1}^{s}P_{i}^{2}}$$

(4) $$J=\frac{H^{\prime }}{H_{\max}},H_{\max}=\ln S$$

where, *S* represents the number of all species in the community; N represents the number of all individuals in the community; and $n_{i}$ represents the number of individuals in the i-th species in the community.

The method of inferring the bacterial functional group is as follows: First, phyla and genera of bacteria were identified by 16S rRNA. Then, the bacteria were classified according to the different functional groups by comparing them with known bacteria and groups summarized in the literature [44,45]. Then, the percentage of each functional group was calculated according to its relative abundance.

Meanwhile, the significance tests were conducted using a two-way ANOVA (Analysis of Variance) method of the SPSS 17.0 software (SPSS, Inc., Chicago, IL, USA). First, normal distribution test was conducted in the original data by the SPSS software. Then, non-normal distribution was found according to the Kolmogorov-Smirnova (KS) parameter. Therefore, data were transformed into logarithm scale (X’ = log (X + 1)) for satisfying the normal distribution, and this formula was identified considering smaller data values. Finally, the homogeneity test of variance was performed before the ANOVA analysis, because it is an important prerequisite for the analysis of variance and F test, which is to test whether the variance of two samples is the same. Then, multiple comparisons were done by a post hoc test, which is the procedure referred to as the Least Significant Difference (LSD) in SPSS. At the same time, F values of the two-way ANOVA are shown in the figures. Bacterial differences in controls and treatments are performed using DESeq2 (Version 1.20.0, http://www.bioconductor.org/packages/) on Majorbio I-Sanger Cloud Platform. Furthermore, the correlation of bacterial community with the soil parameters is evaluated using RDA (redundancy analysis) by Canoco for Windows version 5.0 [46]. The corresponding analysis of bacterial (at the genus level) and soil characteristics was performed by Canoco software. First, the data of bacteria (the first 12 selected data) in *S. salsa* treatment groups were transformed by the method of log (x+1) before further analysis. Then, the data were processed by the Detrend Correspondence Analysis (DCA). The maximum values of sorting axis that originated from DCA in P2C1, P2C2, and P2C3 treatments are 1.042, 2.068, and 1.074, respectively. As these values were all lower than 3, the linear model redundancy analysis (RDA) was selected to perform the corresponding analysis.

## 3. Results and Discussion

### 3.1. Physiochemical Characteristics of the Soil

**Figure 1:**
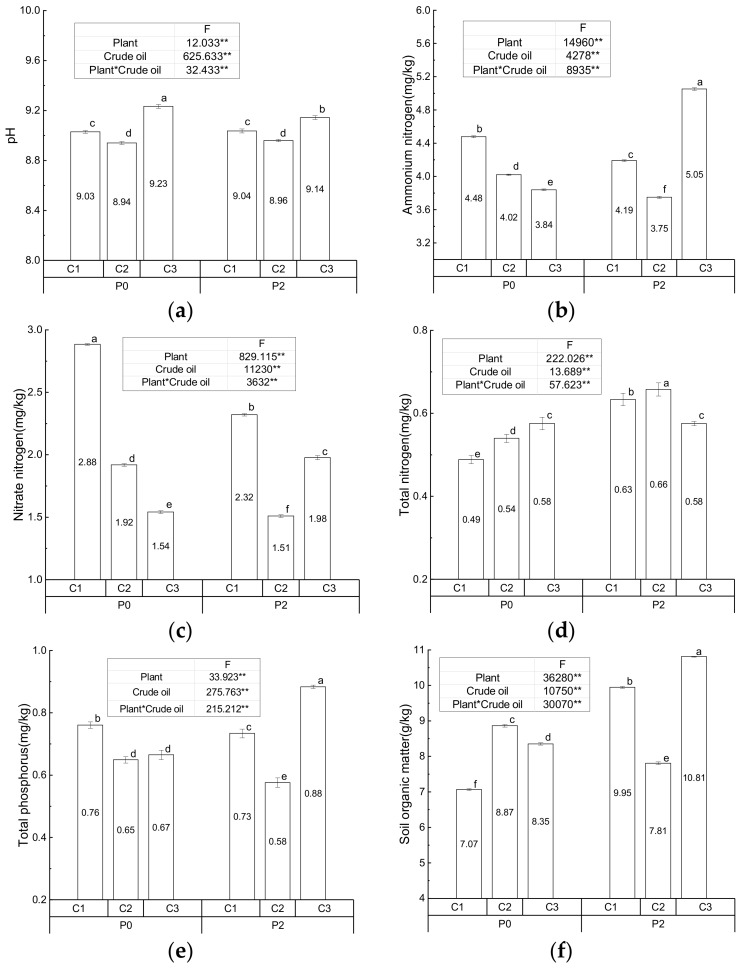
Soil physiochemical composition, including pH (**a**), Ammonium nitrogen (**b**), nitrate nitrogen (**c**), Total nitrogen (**d**), Total phosphorus (**e**), Soil organic matter (**f**) in all controls and treatments. (** indicates the significant difference at the 0.01 level, and average values are in the center of column (calculated from three replicates), meanwhile the error bar of the column is the value of standard deviation. Columns noted by the same and different small letter means insignificant and significant difference at the 0.05 level, respectively.).

The pH, NH_4_-N, NO_3_-N, TN, TP, and SOM of the control and treatment groups are given in Figure 1. Significant effects of *S. salsa*, crude oil, and their interaction with on the soil parameters were found according to F values and significance level. *S. salsa* significantly increased the NH_4_-N, NO_3_-N, TP, and SOM in the high crude oil-contaminated soil, TN in the low and medium ones, and SOM in the low ones. However, *S. salsa* remarkably reduced the NH_4_-N, NO_3_-N, and TP in the low and medium crude oil-contaminated soil, and pH in the high one. In the absence of *S. salsa, an* elevated crude oil concentration significantly increased the TN content, and remarkably reduced NH_4_-N and NO_3_-N. Significant differences in pH, TP, and SOM among soils with three crude oil concentrations were also found. In the treatment groups with *S. salsa*, the order of significant difference of pH, NH_4_-N, TP, and SOM at three crude oil levels was the same (High > Low > Medium). NO_3_-N (Low > High > Medium) and TN (Medium > Low > High) at three crude oil levels were also significantly different, but the order was different.

The growth of *S. salsa* in the low and medium contamination group was much better than that in the high one, which also could be ascertained by the biomass in the harvest period. Meanwhile, NH_4_-N and NO_3_-N were the main species that could be assimilated by *S. salsa*. Crude oil degradation was accelerated by *S. salsa* and microorganisms. Therefore, phosphorus was gradually released, leading to the increase in phosphorus content. Meanwhile, *S. salsa* was inhibited in the high crude oil-contaminated soil treatment group. For example, plant growth was significantly decreased in crude oil-contaminated soil (46.8 g/kg) than in uncontaminated soil [39]. Significant variations in nitrogen species and phosphorus might be due to the joint effect of release and assimilation. It was reported that crude oil with a high content of nitrogen (0.5%) increased the organic nitrogen in polluted soil in the Shengli Oilfield in China [39]. As a result, significant TN increase in the low and medium contamination groups could be explained by the effect of *S. salsa* and/or microbe. It might have been caused by the nitrogen fixation by non-symbiotic bacteria stimulated by the anaerobic conditions in oil-saturated soil. However, these effects could be inhibited by high crude oil contamination of the soil. A field study showed that crude oil contamination could significantly increase the total organic carbon (TOC) as compared with that of unpolluted soil [47]. A similar increase (from 0.22 to 1.14%) in TOC was also found in polluted soils around oil wells [39]. Therefore, the increase of TOC may be caused by the input of crude oil with the abundant carbon. Only pH in the high contamination group was found to have clearly decreased by *S. salsa*. The same effect of *S. salsa* was found in another pot experiment with crude oil contamination of 1, 2 and 4 g/kg [48]. The decrease in pH might be due to the hydroxamic substances produced by the microbial degradation of petroleum hydrocarbons in the rhizosphere [49]. The crude oil content increased the pH value, which was found to be higher than the background value (8.08) in the wetland [39]. In other studies, it was found that long term contamination of soil with crude oil (9.2 g/kg) led to an increase in the pH from acidic to alkaline levels in surface soils [47], and that increased crude oil contamination could decrease the content of NH_4_-N and NO_3_-N in soils [48].

**Figure 2:**
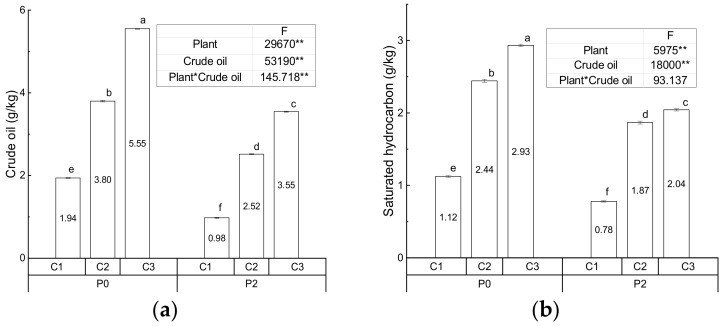

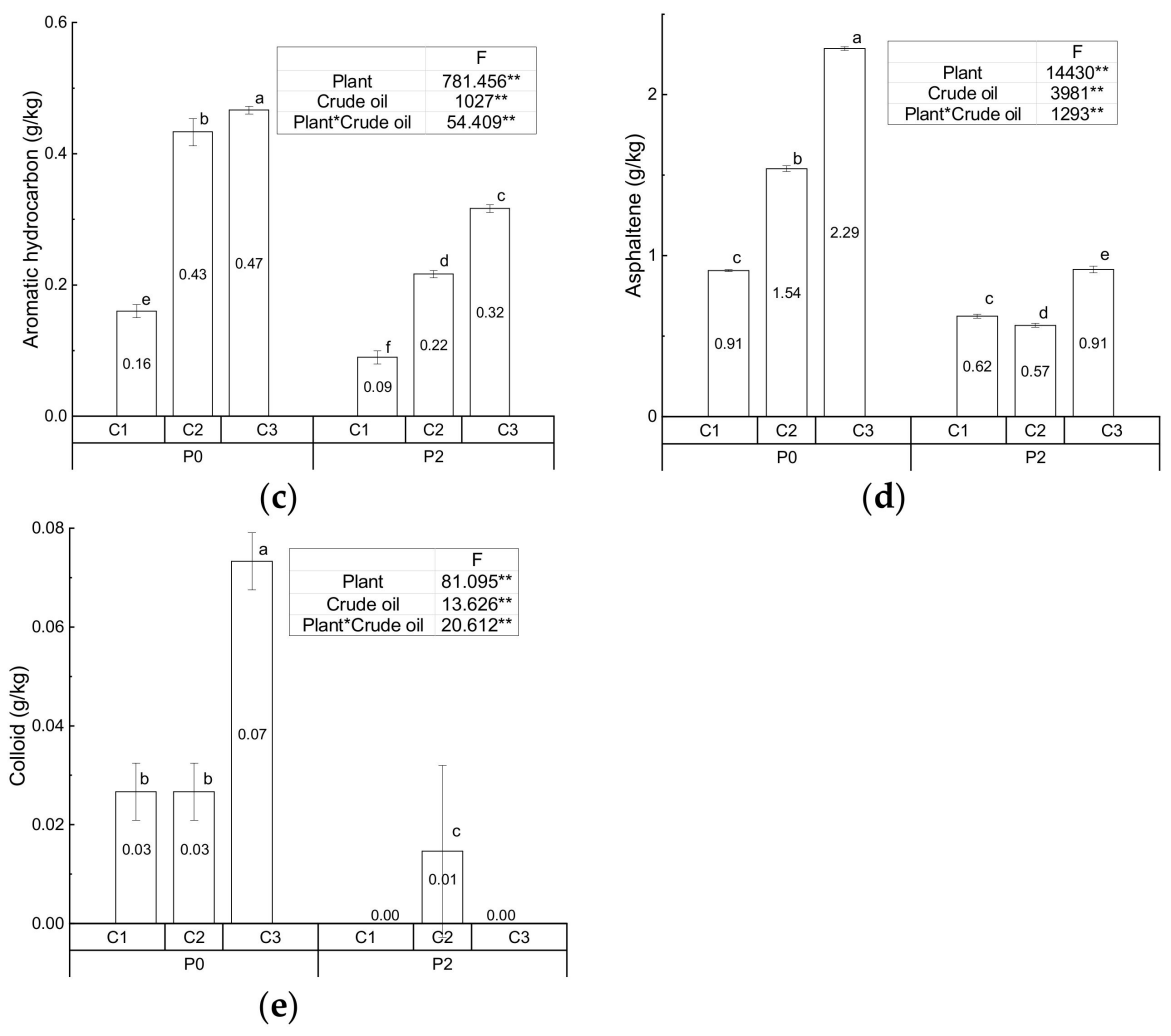
The crude oil (**a**), saturated hydrocarbon (**b**), aromatic hydrocarbon (**c**), asphaltene (**d**) and colloid (**e**) in controls and treatments. (** indicates the significant difference at the 0.01 level, and average values are in the center of column (calculated from three replicates), meanwhile the error bar of the column is the value of standard deviation. Columns noted by the same and different small letter means insignificant and significant difference at the 0.05 level, respectively. Colloid data in the low and high contamination with *S. salsa* are below the detection limit.).

Figure 2 presents the components of crude oil in the control and treatment groups. Impact of *S. salsa*, crude oil, and their interaction are significant. *S. salsa* significantly reduced the contents of crude oil, saturated hydrocarbon, aromatic hydrocarbon, asphaltene, and colloid in soil contaminated with crude oil at three levels with the exception of asphaltene in the low crude oil-contaminated soil. In the three control groups, an increase in the crude oil contamination of the soil clearly increased the components of crude oil except for the content of colloid in the low and medium contamination groups. In the treatment groups with *S. salsa*, the contents of crude oil, saturated and aromatic hydrocarbons increased with increased crude oil contamination. The order of asphaltene content at the three levels of contamination is, medium < low < high. Meanwhile, the colloid content in the low and high contamination group with *S. salsa* was below the detection limit. Very minor reduction in the crude oil content in the three controls with different crude oil contents was found, which is 3, 5, and 2.5%, respectively, for low, medium, and high contamination groups. This result could be explained by natural degradation. Natural degradation of 12% crude oil was found in the case of contaminated soil with no vegetation in another study [21]. In order to calculate the degradation of crude oil caused by *S. salsa* treatment, the reduction of the crude oil caused in controls should be deducted. As a result, degradation rates of crude oil, saturated hydrocarbon, aromatic hydrocarbon, asphaltene, and colloid in the low contamination treatment group are calculated as 47.16, 28.30, 35.66, 17.76, and 100%, respectively; the corresponding degradation rates in the medium contamination treatment group are 30.50, 22.67, 47.54, 47.28, and 100%, respectively; and the corresponding ones in the high contamination treatment group are 32.38, 19.49, 31.40, 43.80, and 28.18%, respectively. The degradation of aromatic hydrocarbons was higher than that of saturated hydrocarbons. Note that this value is just for September and not for the whole growth period. The reason for this difference could be that the abundance of rhizosphere microbes or bacteria specifically used to degrade aromatic hydrocarbons might have been different. Some unknown specific microbe might have played an important role, while the rhizosphere of *S. salsa* provided the living environment. The degradation of crude oil and saturated hydrocarbons was higher in the low contamination treatment group than in others, while the degradation of the aromatic hydrocarbons and asphaltene in the medium contamination treatment group was the highest. A higher reduction (92%) in the content of aromatic hydrocarbons was also observed in the treatment of contaminated soil with *Vertiveria zizanioides* and microbes by Dhote et al. [11]. A much higher degradation of crude oil in contaminated soil by *Leptochloa fusca* (51%) and *Brachiaria mutica* (61%) was reported by Fatima et al. [21]. Further, different degradation mechanisms of plants, such as uptake oil from soil and transformation inside the plant, have been reported [50,51]. Furthermore, it has been reported that combining plant and bacteria results in higher degradation of crude oil, i.e., in the range of 78% to 85% [21] and 85% [11]. In addition, It was found that fungi could also play a role in the degradation of hydrocarbons, and the synergistic action between fungi and plants, such as *Megathyrsus maximus,* accelerates the degradation of hydrocarbons [52].

### 3.2. Diversity of the Bacterial Community

**Figure 3:**
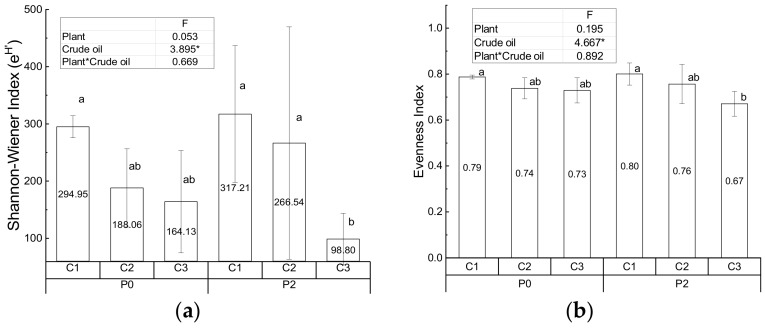
Exponential Shannon-Wiener index (**a**) and Evenness Index (**b**) of controls and treatments. (* indicates the significant difference at the 0.05 level, and average values are in the center of column (calculated from three replicates), meanwhile the error bar of the column is the value of standard deviation. Columns noted by the same and different small letter means insignificant and significant difference at the 0.05 level, respectively).

The Exponential Shannon-Wiener index and Evenness index of control and treatment groups are presented in Figure 3. Good’s coverage is higher than 98%. No significant difference of Simpson’s Reciprocal index among controls and treatment groups (P0C1: 78.16 ± 10.94; P0C2: 57.51 ± 21.10; P0C3: 58.18 ± 32.51; P2C1: 103.98 ± 89.11; P2C2: 97.97 ± 105.08; P2C3: 29.86 ± 15.56) are found by F test. The F values of plant, crude oil and interaction of plant with crude oil are 0.222, 1.815 and 0.665, respectively, but their p values are all higher than 0.05. Crude oil significantly influences the Exponential Shannon-Wiener index and Evenness index, while the effect of *S. salsa* and its interaction with crude oil are not prominent. In the treatment groups with *S. salsa*, the difference in the Exponential Shannon-Wiener index in the low and medium contamination group is not significant; the Exponential Shannon-Wiener indexes of both are however significantly higher than that of the high contamination group. In addition, the Evenness index of the low contamination group with *S. salsa* is significantly higher than that of the high contamination one. It has been shown that the bacterial diversity in soil is remarkably reduced by oil contamination, regardless of the soil matrix type [53]. Similar results i.e., a decline in the overall microbial diversity owing to contamination has been documented in the literature [54,55]. Therefore, the impact of high crude oil contamination on the bacterial diversity is greater than the effect of plant [56], which may be used to explain the decline in the bacterial diversity in the high contamination treatment group.

In addition, the significant decrease in the Evenness index of the high contamination group with *S. salsa* could be due to the abundance of degradation bacteria selectively stimulated by the crude oil contaminants [57]. Fungi such as *Megathyrsus maximus* could accelerates the degradation of hydrocarbons [52]. Meanwhile, bacteria were found to be more versatile than fungi in degrading the petroleum hydrocarbons [58,59], and the hydrocarbon degradation could be increased by the root activity of alfalfa and perennial ryegrass [52]. Therefore, *S. salsa* could promote microbial diversity under low and medium crude oil contamination, but high crude oil pollution played a leading role in determining the microbial diversity. The dynamic synergy between plant roots and rhizosphere bacteria should be studied further. Bacteria increased the plant’s resistance to contaminant stress and enhanced the plant biomass, while the plant supported microbial degradation and mineralization of hydrocarbons through their rhizosphere effects [60,61].

The Exponential Shannon-Wiener index and Evenness Index data in controls and treatment are organized in SPSS software according to the levels of crude oil contamination. Further analysis is performed to identify the difference among three crude oil levels. Only the Exponential Shannon Wiener Index and Evenness Index in low crude oil contamination (306.08 ± 77.68; 0.80 ± 0.03) are significantly higher than that in high contamination (131.46 ± 29.70; 0.75 ± 0.06). It furtherly indicates that crude oil contamination is the key impacting factor for bacteria.

### 3.3. Taxonomic Compositions

According to the OTU-based analysis, the individual sequences were grouped into 23 phyla and 92 genera in soil (Figure S1, Tables S1 and S2). At the phylum level, the soil bacteria in the low contamination control group were dominated by *Proteobacteria* (50.33%), *Chloroflexi* (14.33%), and *Acidobacteria* (13.00%), and dominant bacteria including *Proteobacteria* (35.33%), *Chloroflexi* (13.33%) and *Actinobacteria* (12.67%), *Saccharibacteria* (12.00%), *and Acidobacteria* (12.67%) were found in the low contamination group with *S. salsa*. The bacteria in the medium contamination group were dominated by *Proteobacteria* (43.67%), *Chloroflexi* (14.67%), and *Acidobacteria* (12.67%), while *Proteobacteria* (52.67%) and *Chloroflexi* (11.33%) were prevalent in the medium contamination treatment group. Meanwhile, the predominant bacteria in the high contamination control were *Proteobacteria* (36.67%) and *Acidobacteria* (17.33%). Further, the dominant bacteria in the high contamination group with *S. salsa* were *Proteobacteria* (47.00%) and *Saccharibacteria* (13.33%). *Proteobacteria* were always the dominant ones among all control and treatment groups.

At the genus level, the top 12 most prevalent bacteria were counted, and their percentage was found to be 49, 48, 48, 41, 47, and 50% in the low, medium, and high contamination control and treatment groups, respectively. The dominant bacteria in the low contamination control group were *Anaerolineaceae_uncultured* (11%), *Moraxellaceae _uncultured* (5%), *Desulfuromonas* (5%), *Pseudomonas* (5%), and *C1-B045* (5%), while those in the low contamination group with *S. salsa* were *Anaerolineaceae_uncultured* (9%), *Saccharibacteria* (12%), and *Pseudomonas* (6%). The dominant bacteria in the medium contamination control group were *Anaerolineaceae_uncultured* (11%), *Desulfuromonas* (5%), and *Saccharibacteria* (5%), while those in the medium contamination group with *S. salsa* were *Anaerolineaceae_uncultured* (7%) and *Alcanivorax* (9%). Further, the dominant bacteria in the high contamination control were *Saccharibacteria* (8%) and *Mycobacterium* (8%), while those in the high contamination with *S. salsa* were *Desulfuromonas* (9%), *PYR10d3* (6%), and *Cytophagaceae _uncultured* (5%). *Anaerolineaceae_uncultured* were the prevalent bacteria in the low and medium contamination treatment groups, whereas they were not the dominant ones in the high treatment group.

The dominant phylum in three controls was the same except *Chloroflexi* in high contamination. And the *Chloroflexi* might be inhibited by high crude oil contamination. While, no significant differences of bacteria were found between controls and corresponding treatment group (Figures S2 and S3). It showed that *S. salsa* did not significantly affect the abundance of a certain bacteria, although the *Actinobacteria* and *Saccharibacteria* increased in low and medium contamination treatment with *S. salsa*; the *Alcanivorax* increased in the medium contamination treatment; the *Saccharibacteria* and *Acidobacteria* increased and decreased in the high treatment group as compared with the control group, respectively. In addition, new ones including *PYR10d3*, and *Cytophagaceae _uncultured* were observed in the high contamination treatment group.

Sutton et al. [53] found that *Proteobacteria,* *Firmicutes, Actinobacteria, Acidobacteria,* and *Chloroflexi* were the major microbiota in long-term diesel-contaminated soils. Further, the work of Peng et al. [57] showed that *Actinomycetes,* *Bacteroidetes, Chloroflexi, Planctomycetes*, *and Proteobacteria* were the major bacteria in crude oil-contaminated soil. Most of the bacteria found in other studies, such as *Proteobacteria, Chloroflexi, and Acidobacteria,* were also found in our study. Among crude oil degradation bacteria, *Alcanivorax* specifically degraded monocyclic aromatic compounds, while *Pseudomonas* degraded aliphatic and polycyclic aromatic hydrocarbons [14,27]. Crude oil contains various compounds, and no single bacteria could degrade all components, because an individual bacterium could only degrade a narrow range of hydrocarbons [62,63]. Therefore, several kinds of bacteria usually appeared at the same time, which were for different petroleum components.

### 3.4. Functional Group Distribution

**Figure 4:**
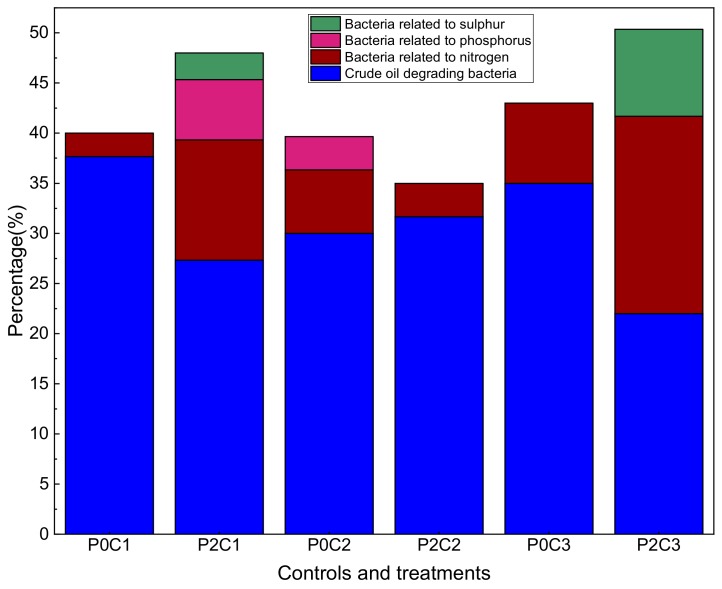
Functional groups distribution in the controls and treatments.

Bacteria performing certain functions in the control and treatment groups (Figure 4) were inferred by the results from literature [44,64]. The largest percentage of the bacteria in the experimental group was the crude oil degradation bacteria, which was found at 37.67, 27.33, 30.00, 31.67, 35.00, and 22.00% in the low, medium, and high contamination control and treatment groups, respectively. Compared with those in the low crude oil contamination control group, *S. salsa* decreased the percentage of crude oil degradation bacteria, while increasing the percentage of sulfur, phosphorus, and nitrogen-related bacteria. Meanwhile, opposite and similar results were found in the medium and high treatment groups, respectively. The P cycling bacteria were not found in the medium and high contamination group with *S. salsa*, which indicated their strong inhibition by the medium and high crude oil contamination. S cycling bacteria were absent in control groups, but were found in the high contamination group with *S. salsa* (8.67%) at a higher abundance than that in the low contamination treatment group. This result possibly indicates that this metabolism is related to the S composition of the crude oil. However, the opposite result was obtained for the medium contamination group with *S. salsa*. Meanwhile, P cycling bacteria were only found in the low contamination group with *S. salsa* (6.00%) and the medium contamination control group (3.33%). This may be due to the uncertainty of bacterial function owing to the limited literature.

### 3.5. Relationship between Bacterial Groups and Soil Variables

**Figure 5:**
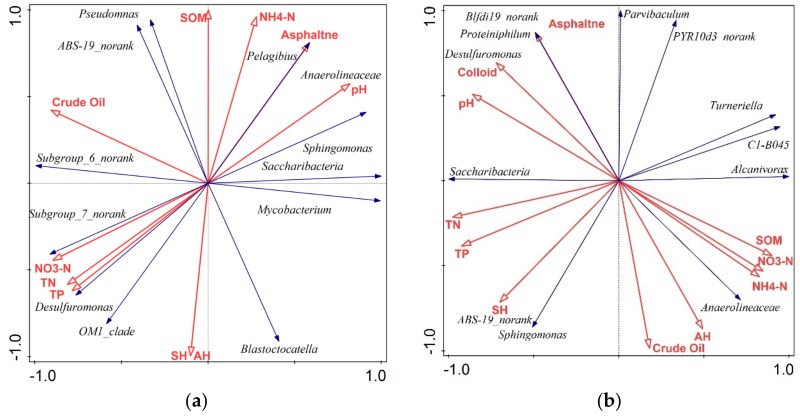

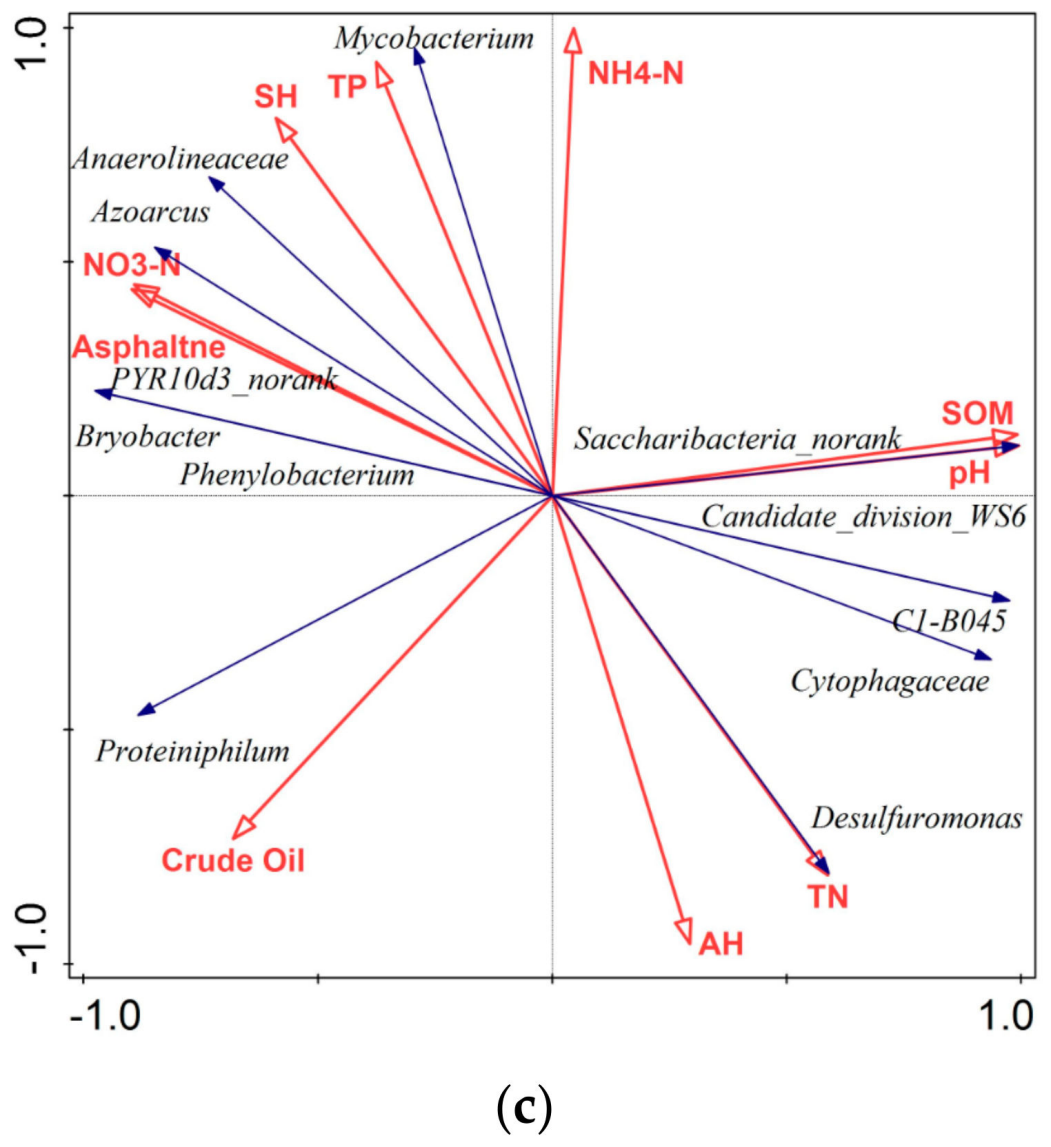
The RDA of bacteria and environmental factors in P2C1 (**a**), P2C2 (**b**), and P2C3 (**c**) treatments (SH and AH are the abbreviations for saturated and aromatic hydrocarbon, respectively).

Linear model redundancy analysis (RDA) of bacteria and environmental factors in *S. salsa* treatment groups is presented in Figure 5. And the correlations of bacteria with environmental parameters in P2C1, P2C2 and P2C3 treatments are given in Tables S3, S4 and S5, respectively.

Bacterial variables are explained by the eigenvalues (horizontal axis), with percentages of 84.2%, 81.8%, and 93.5% in three treatment groups with *S. salsa*, which indicated the good ranking results. In the low crude oil-contaminated soil with *S. salsa*, *Anaerolineaceae_uncultured* (9.33%) belonging to the crude oil degradation bacteria has a negative relationship with the crude oil and saturated/aromatic hydrocarbon. In addition, no significant correlation of other crude oil degradation bacteria with the environmental factors was found. *Saccharibacteria* belonging to bacteria related to N cycle has a positive correlation with pH, asphaltene, NH_4_-N and SOM, and *Pseudomonas* belonging to bacteria with P cycle is positively related to SOM, NH_4_-N, asphaltene, pH, and crude oil. All these correlations are not significant. However, the correlation of *Anaerolineaceae* and *Pelagibius* with asphaltene, *Subgroup_7* with NO_3_-N, is significant. *Desulfuromonas* belonging to bacteria related to S cycle has a significant positive correlation with TN and TP, while having a negative correlation with pH. In the medium crude oil-contaminated soil with *S. salsa*, most bacteria belonging to crude oil degradation are negatively correlated to crude oil concentration except for *Anaerolineaceae_uncultured* (7%). *C1-B045* (2.33%) and *Turneriella* (2.33%) both have significant and negative correlations with TN and TP. The correlation of *Anaerolineaceae_uncultured* (7%) with colloid, and correlation of *Parvibaculum* (2.0%) with crude oil are both significant and negative. Meanwhile, *Proteiniphilum* (1.67%) has a significant negative correlation with aromatic hydrocarbons and a significant positive correlation with asphaltene. *Saccharibacteria* (3.33%) of N cycling bacteria has a positive correlation with TN and TP, and a negative correlation with NH_4_-N and NO_3_-N, both of which are not significant. In the high crude oil-contaminated soil with *S. salsa*, *Desulfuromonas* (8.67%) related to the S cycle showed a remarkable and negative correlation with saturated hydrocarbons, and significant and positive correlation with TN. Meanwhile, *Azoarcus* (2.67%) related to the N cycle showed a significant positive correlation with NO_3_-NThe correlation of the crude oil degradation bacteria with crude oil/oil components and nitrogen is mostly significant and negative, indicating the significant effect of crude oil on the related degradation bacteria. A similar effect was also observed in other studies [56,57]. Meanwhile, both negative correlations were caused by crude oil in Shengli Oilfield with a high nitrogen content. However, a clear difference was found in previous studies, which showed that pH is an important driver of bacterial community [65,66]. Significant correlation of bacteria related to the N cycle with different nitrogen species was found among the three treatment groups. Furthermore, the positive or negative correlation was caused by different nitrogen transformations [67,68,69]. Significant and positive correlation of P cycling bacteria with crude oil and nitrogen species was found only in the low contamination treatment group. In addition, significant correlation of S cycling bacteria with crude oil components was found in the medium and high treatment groups. This result might be closely associated with the phosphorus content [67] and *S. salsa* [70]. Most crude oil degradation bacteria are negatively related to crude oil content. While, the relationship of other bacteria with crude oil or physio-chemical properties varies greatly depending on different parameters. N cycling bacteria were sensitive to TN, TP, ammonia nitrogen, nitrate nitrogen, and pH.

## 4. Conclusions

Effects of *S. salsa*, crude oil, and their interaction on the physiochemical parameters of the soil and crude oil degradation were significant. The effects differed among the control and treatment groups. *Proteobacteria* were dominant among all control and treatment groups at the phylum level. At the genus level, *Saccharibacteria* and *Alcanivorax* were found to increase in the low and medium contamination treatment groups, respectively. Meanwhile, *Desulfuromonas*, *PYR10d3,* and *Cytophagaceae _uncultured* were prevalent in the high contamination treatment group. Crude oil significantly influenced the bacterial diversity, while the effect of *S. salsa* and its interaction with crude oil were not significant. The bacteria related to crude oil degradation were the largest percentage. *S. salsa* reduced crude oil bacteria and increased the abundance of bacteria related to S, P, and N cycles in the low and high contamination treatment groups, while the contrary results were obtained for the medium contamination treatment group. Most crude oil degradation bacteria showed negative correlation with the crude oil content. Bacteria related to nitrogen cycle were sensitive to TN, TP, NH_4_-N, NO_3_-N, and pH of soil. Further, bacteria related to S cycle were sensitive to aromatic hydrocarbons, saturated hydrocarbons, and asphalt in soil. Microbial community related to N, P, and S cycle would be changed by crude oil contamination, that may cause the changes of major element cycle. Studies have shown that degradation of crude oil contamination by in situ was an effective measure. Combination of bacteria, fungi, and others with plants such as S. salsa would be more effective for degradation considering the existing literature, although fungi were not involved in this study. In the future, we plan to carry out a similar study on soil contaminated with crude oil at concentrations below 2 g/kg and above 6 g/kg.

## Supplementary Materials

The following are available online at https://www.mdpi.com/1660-4601/17/5/1471/s1, Figure S1: Phylum (a) and genus (b) compositions of controls and Suaeda treatments. The sequences with relative abundance <1% which could be classified into known groups were assigned to “others”. Figure S2: Difference of bacteria at phylum level between the controls and treatments of low (a), medium (b) and high (c) contamination. Figure S3: Difference of bacteria at genus level between the controls and treatments of low (a), medium (b) and high (c) contamination. Table S1: Fraction of phylum composition in controls and treatments. Table S2: Fraction of genus composition in controls and treatments. Table S3: Correlation of bacteria and environmental parameters in the low contamination treatment with *S. salsa* (P2C1). Table S4: Correlation of bacteria and environmental parameters in the medium contamination treatment with *S. salsa* (P2C2). Table S5: Correlation of bacteria and environmental parameters in the high contamination treatment with *S. salsa* (P2C3).
