# Supplementary material for: Remediation of Crude Oil-Polluted Soil by the Bacterial Rhizosphere Community of Suaeda Salsa Revealed by 16S rRNA Genes
Source: Int J Environ Res Public Health. 2020 Feb 25;17(5):1471. doi: 10.3390/ijerph17051471 (PMC7084840; doi:10.3390/ijerph17051471)
Supplement: Supplementary file 1 [file ijerph-17-01471-s001.zip › IJERPH-663989-SI/ijerph-663983- Figure S1-S3.docx]

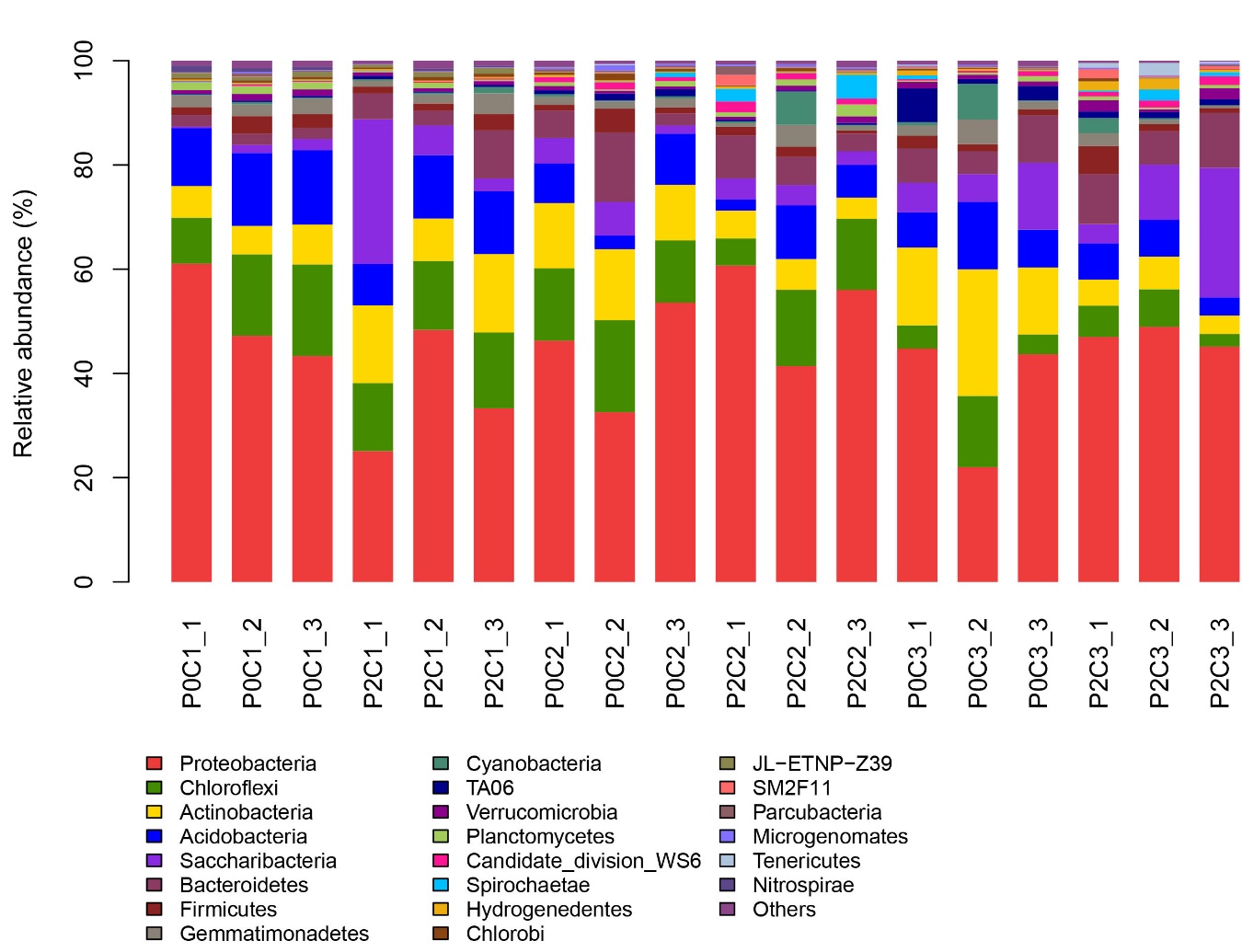


（a）


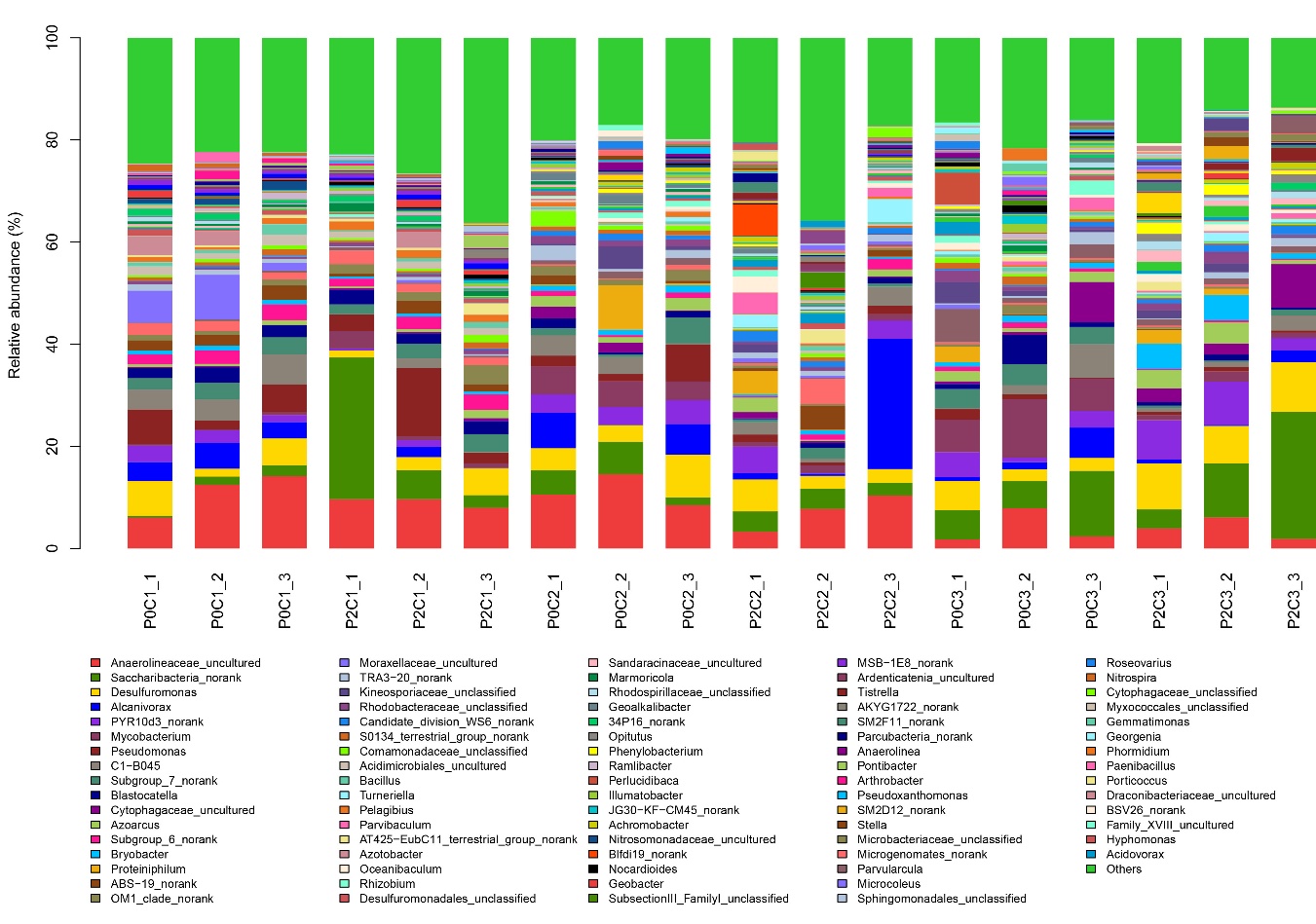


(b)

**Figure S1.** Phylum (a) and genus (b) compositions of controls and Suaeda treatments. The sequences with relative abundance <1% which could be classified into known groups were assigned to “others”.


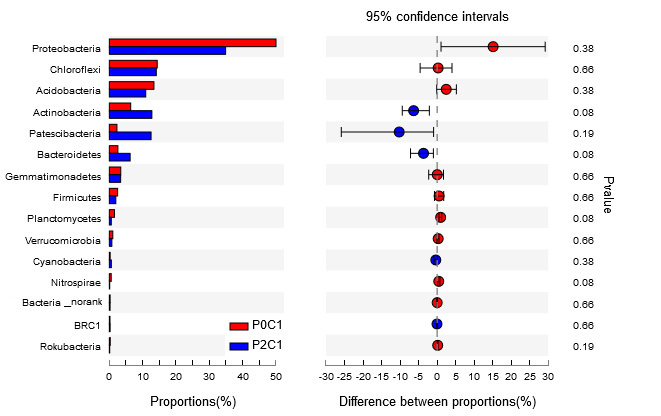


(a)


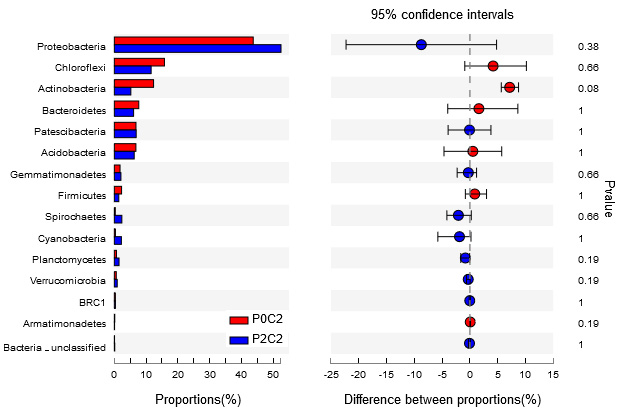


(b)


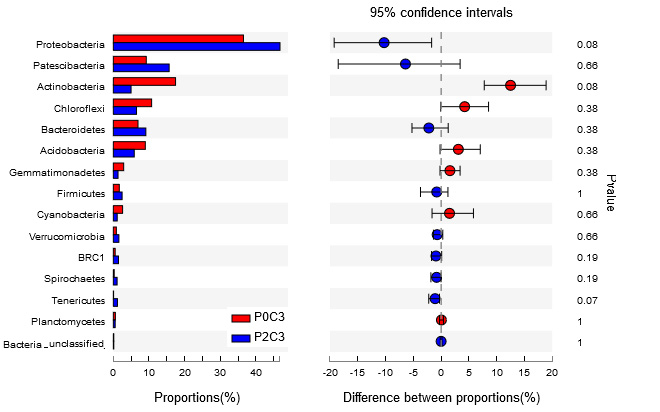


(c)

**Figure S2.** Difference of bacteria at phylum level between the controls and treatments of low (a), medium (b) and high (c) contamination.


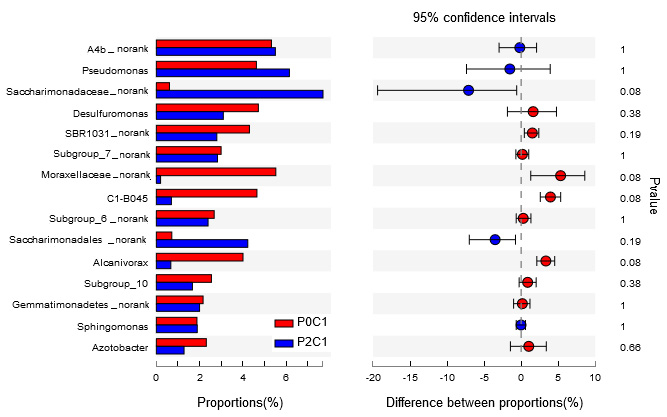


(a)


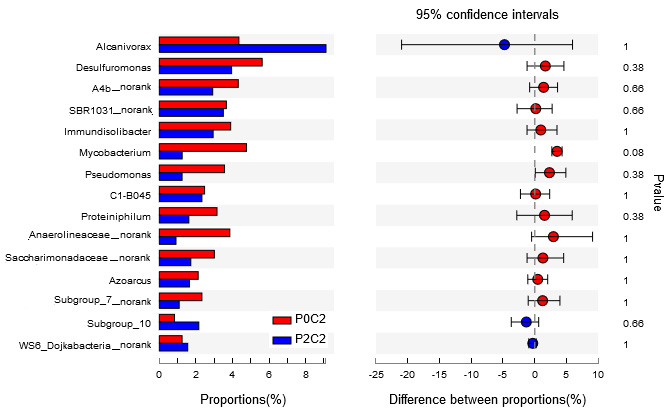


(b)


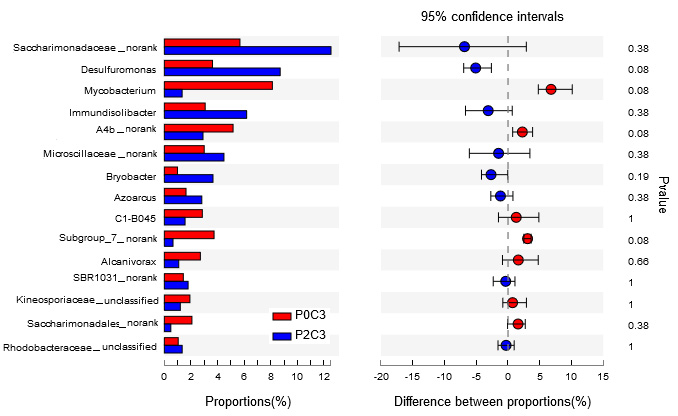


(c)

**Figure S3.** Difference of bacteria at genus level between the controls and treatments of low (a), medium (b) and high (c) contamination.
