# Supplementary material for: Remediation of Crude Oil-Polluted Soil by the Bacterial Rhizosphere Community of Suaeda Salsa Revealed by 16S rRNA Genes
Source: Int J Environ Res Public Health. 2020 Feb 25;17(5):1471. doi: 10.3390/ijerph17051471 (PMC7084840; doi:10.3390/ijerph17051471)
Supplement: Supplementary file 1 [file ijerph-17-01471-s001.zip › IJERPH-663989-SI/ijerph-663983-Table S1-S5.docx]

|  | P0C1_1 | P0C1_2 | P0C1_3 | P2C1_1 | P2C1_2 | P2C1_3 | P0C2_1 | P0C2_2 | P0C2_3 | P2C2_1 | P2C2_2 | P2C2_3 | P0C3_1 | P0C3_2 | P0C3_3 | P2C3_1 | P2C3_2 | P2C3_3 |
| --- | --- | --- | --- | --- | --- | --- | --- | --- | --- | --- | --- | --- | --- | --- | --- | --- | --- | --- |
| Proteobacteria | 0.611 | 0.472 | 0.434 | 0.251 | 0.484 | 0.333 | 0.463 | 0.326 | 0.536 | 0.607 | 0.414 | 0.560 | 0.447 | 0.220 | 0.437 | 0.470 | 0.489 | 0.451 |
| Chloroflexi | 0.088 | 0.156 | 0.176 | 0.130 | 0.132 | 0.146 | 0.139 | 0.176 | 0.119 | 0.052 | 0.146 | 0.137 | 0.045 | 0.136 | 0.038 | 0.060 | 0.072 | 0.024 |
| Actinobacteria | 0.061 | 0.055 | 0.076 | 0.150 | 0.082 | 0.150 | 0.125 | 0.137 | 0.107 | 0.054 | 0.059 | 0.040 | 0.149 | 0.243 | 0.128 | 0.051 | 0.063 | 0.036 |
| Acidobacteria | 0.111 | 0.140 | 0.143 | 0.079 | 0.121 | 0.121 | 0.076 | 0.026 | 0.098 | 0.021 | 0.103 | 0.063 | 0.067 | 0.129 | 0.073 | 0.069 | 0.071 | 0.035 |
| Saccharibacteria | 0.003 | 0.016 | 0.022 | 0.278 | 0.057 | 0.025 | 0.049 | 0.064 | 0.016 | 0.040 | 0.039 | 0.026 | 0.057 | 0.053 | 0.128 | 0.037 | 0.106 | 0.249 |
| Bacteroidetes | 0.022 | 0.021 | 0.021 | 0.049 | 0.028 | 0.092 | 0.053 | 0.133 | 0.023 | 0.083 | 0.054 | 0.034 | 0.066 | 0.044 | 0.091 | 0.096 | 0.064 | 0.105 |
| Firmicutes | 0.015 | 0.034 | 0.027 | 0.013 | 0.014 | 0.032 | 0.011 | 0.046 | 0.012 | 0.016 | 0.019 | 0.006 | 0.024 | 0.014 | 0.011 | 0.054 | 0.014 | 0.010 |
| Gemmatimonadetes | 0.023 | 0.022 | 0.030 | 0.012 | 0.017 | 0.040 | 0.016 | 0.014 | 0.018 | 0.007 | 0.042 | 0.009 | 0.020 | 0.047 | 0.015 | 0.025 | 0.008 | 0.004 |
| Cyanobacteria | 0.002 | 0.005 | 0.001 | 0.003 | 0.004 | 0.012 | 0.004 | 0.002 | 0.004 | 0.003 | 0.064 | 0.002 | 0.006 | 0.069 | 0.002 | 0.029 | 0.003 | 0.001 |
| TA06 | 0.002 | 0.003 | 0.004 | 0.006 | 0.002 | 0.004 | 0.008 | 0.013 | 0.014 | 0.003 | 0.001 | 0.004 | 0.065 | 0.009 | 0.028 | 0.013 | 0.012 | 0.012 |
| Verrucomicrobia | 0.007 | 0.012 | 0.013 | 0.007 | 0.007 | 0.008 | 0.008 | 0.005 | 0.005 | 0.006 | 0.010 | 0.012 | 0.012 | 0.008 | 0.009 | 0.022 | 0.005 | 0.021 |
| Planctomycetes | 0.015 | 0.015 | 0.014 | 0.004 | 0.010 | 0.003 | 0.007 | 0.003 | 0.010 | 0.009 | 0.012 | 0.023 | 0.003 | 0.004 | 0.010 | 0.007 | 0.003 | 0.006 |
| Candidate_division_WS6 | 0.001 | 0.003 | 0.002 | 0.001 | 0.002 | 0.002 | 0.010 | 0.014 | 0.008 | 0.021 | 0.012 | 0.011 | 0.003 | 0.003 | 0.010 | 0.009 | 0.014 | 0.017 |
| Spirochaetae | 0.001 | 0.000 | 0.001 | 0.000 | 0.000 | 0.000 | 0.000 | 0.001 | 0.009 | 0.024 | 0.000 | 0.046 | 0.007 | 0.000 | 0.000 | 0.003 | 0.021 | 0.008 |
| Hydrogenedentes | 0.003 | 0.003 | 0.002 | 0.002 | 0.003 | 0.003 | 0.004 | 0.003 | 0.002 | 0.005 | 0.003 | 0.004 | 0.009 | 0.004 | 0.002 | 0.018 | 0.021 | 0.004 |
| Chlorobi | 0.003 | 0.005 | 0.005 | 0.003 | 0.007 | 0.006 | 0.004 | 0.013 | 0.005 | 0.002 | 0.006 | 0.002 | 0.003 | 0.001 | 0.001 | 0.006 | 0.002 | 0.001 |
| JL-ETNP-Z39 | 0.009 | 0.006 | 0.009 | 0.005 | 0.009 | 0.011 | 0.001 | 0.001 | 0.002 | 0.000 | 0.001 | 0.002 | 0.001 | 0.001 | 0.000 | 0.000 | 0.001 | 0.000 |
| SM2F11 | 0.000 | 0.001 | 0.000 | 0.000 | 0.000 | 0.000 | 0.001 | 0.002 | 0.001 | 0.020 | 0.001 | 0.001 | 0.002 | 0.001 | 0.001 | 0.017 | 0.002 | 0.006 |
| Parcubacteria | 0.001 | 0.006 | 0.002 | 0.000 | 0.002 | 0.000 | 0.005 | 0.003 | 0.003 | 0.016 | 0.002 | 0.003 | 0.002 | 0.002 | 0.004 | 0.000 | 0.000 | 0.003 |
| Microgenomates | 0.000 | 0.003 | 0.002 | 0.001 | 0.001 | 0.001 | 0.003 | 0.010 | 0.004 | 0.003 | 0.004 | 0.002 | 0.002 | 0.003 | 0.000 | 0.003 | 0.002 | 0.002 |
| Tenericutes | 0.000 | 0.000 | 0.000 | 0.000 | 0.000 | 0.000 | 0.000 | 0.002 | 0.000 | 0.000 | 0.000 | 0.000 | 0.002 | 0.000 | 0.000 | 0.009 | 0.024 | 0.004 |
| Nitrospirae | 0.012 | 0.008 | 0.005 | 0.001 | 0.004 | 0.003 | 0.001 | 0.000 | 0.000 | 0.000 | 0.001 | 0.000 | 0.000 | 0.000 | 0.000 | 0.000 | 0.000 | 0.000 |
| Others | 0.010 | 0.014 | 0.012 | 0.006 | 0.015 | 0.010 | 0.012 | 0.006 | 0.006 | 0.007 | 0.006 | 0.013 | 0.007 | 0.008 | 0.010 | 0.004 | 0.004 | 0.002 |

**Table S1.** Fraction of phylum composition in controls and treatments.

**Table S2.** Fraction of genus composition in controls and treatments.

|  | P0C1_1 | P0C1_2 | P0C1_3 | P2C1_1 | P2C1_2 | P2C1_3 | P0C2_1 | P0C2_2 | P0C2_3 | P2C2_1 | P2C2_2 | P2C2_3 | P0C3_1 | P0C3_2 | P0C3_3 | P2C3_1 | P2C3_2 | P2C3_3 |
| --- | --- | --- | --- | --- | --- | --- | --- | --- | --- | --- | --- | --- | --- | --- | --- | --- | --- | --- |
| Anaerolineaceae_uncultured | 0.061 | 0.125 | 0.142 | 0.097 | 0.097 | 0.080 | 0.105 | 0.145 | 0.085 | 0.033 | 0.078 | 0.104 | 0.018 | 0.079 | 0.024 | 0.040 | 0.061 | 0.019 |
| Saccharibacteria_norank | 0.003 | 0.016 | 0.022 | 0.277 | 0.057 | 0.025 | 0.048 | 0.064 | 0.015 | 0.040 | 0.039 | 0.025 | 0.057 | 0.053 | 0.128 | 0.037 | 0.106 | 0.249 |
| Desulfuromonas | 0.068 | 0.015 | 0.052 | 0.014 | 0.025 | 0.052 | 0.043 | 0.032 | 0.083 | 0.063 | 0.026 | 0.027 | 0.058 | 0.024 | 0.025 | 0.091 | 0.073 | 0.097 |
| Alcanivorax | 0.037 | 0.051 | 0.032 | 0.001 | 0.020 | 0.001 | 0.069 | 0.001 | 0.060 | 0.013 | 0.004 | 0.255 | 0.007 | 0.014 | 0.059 | 0.007 | 0.002 | 0.023 |
| PYR10d3_norank | 0.032 | 0.024 | 0.014 | 0.004 | 0.013 | 0.000 | 0.036 | 0.035 | 0.047 | 0.052 | 0.001 | 0.035 | 0.049 | 0.009 | 0.033 | 0.077 | 0.085 | 0.024 |
| Mycobacterium | 0.003 | 0.003 | 0.006 | 0.034 | 0.008 | 0.010 | 0.055 | 0.050 | 0.037 | 0.009 | 0.015 | 0.012 | 0.064 | 0.114 | 0.063 | 0.010 | 0.020 | 0.011 |
| Pseudomonas | 0.068 | 0.017 | 0.054 | 0.032 | 0.134 | 0.021 | 0.021 | 0.014 | 0.072 | 0.015 | 0.005 | 0.017 | 0.020 | 0.010 | 0.002 | 0.006 | 0.009 | 0.005 |
| C1-B045 | 0.038 | 0.041 | 0.060 | 0.001 | 0.019 | 0.000 | 0.040 | 0.033 | 0.003 | 0.024 | 0.008 | 0.037 | 0.003 | 0.018 | 0.066 | 0.008 | 0.010 | 0.029 |
| Subgroup_7_norank | 0.023 | 0.033 | 0.033 | 0.019 | 0.029 | 0.035 | 0.015 | 0.005 | 0.050 | 0.006 | 0.021 | 0.006 | 0.037 | 0.041 | 0.034 | 0.005 | 0.002 | 0.012 |
| Blastocatella | 0.019 | 0.030 | 0.024 | 0.028 | 0.020 | 0.026 | 0.018 | 0.005 | 0.013 | 0.002 | 0.010 | 0.011 | 0.010 | 0.057 | 0.009 | 0.007 | 0.012 | 0.004 |
| Cytophagaceae_uncultured | 0.003 | 0.002 | 0.001 | 0.003 | 0.003 | 0.006 | 0.023 | 0.019 | 0.001 | 0.012 | 0.003 | 0.003 | 0.005 | 0.005 | 0.079 | 0.027 | 0.022 | 0.085 |
| Azoarcus | 0.005 | 0.004 | 0.009 | 0.004 | 0.005 | 0.016 | 0.021 | 0.011 | 0.024 | 0.028 | 0.003 | 0.014 | 0.020 | 0.008 | 0.020 | 0.036 | 0.041 | 0.008 |
| Subgroup_6_norank | 0.019 | 0.027 | 0.031 | 0.015 | 0.025 | 0.031 | 0.010 | 0.004 | 0.011 | 0.003 | 0.010 | 0.021 | 0.009 | 0.012 | 0.007 | 0.002 | 0.004 | 0.002 |
| Bryobacter | 0.008 | 0.009 | 0.009 | 0.005 | 0.006 | 0.005 | 0.010 | 0.011 | 0.014 | 0.004 | 0.008 | 0.003 | 0.008 | 0.012 | 0.010 | 0.050 | 0.050 | 0.010 |
| Proteiniphilum | 0.000 | 0.000 | 0.000 | 0.000 | 0.000 | 0.000 | 0.003 | 0.087 | 0.004 | 0.046 | 0.001 | 0.001 | 0.031 | 0.000 | 0.000 | 0.027 | 0.012 | 0.000 |
| ABS-19_norank | 0.020 | 0.021 | 0.028 | 0.005 | 0.025 | 0.014 | 0.018 | 0.000 | 0.005 | 0.005 | 0.047 | 0.013 | 0.001 | 0.003 | 0.001 | 0.002 | 0.001 | 0.001 |
| OM1_clade_norank | 0.011 | 0.007 | 0.012 | 0.019 | 0.017 | 0.038 | 0.019 | 0.012 | 0.022 | 0.006 | 0.004 | 0.005 | 0.005 | 0.018 | 0.005 | 0.002 | 0.003 | 0.001 |
| Sphingomonas | 0.022 | 0.020 | 0.014 | 0.027 | 0.016 | 0.015 | 0.003 | 0.002 | 0.009 | 0.004 | 0.049 | 0.002 | 0.004 | 0.004 | 0.003 | 0.004 | 0.004 | 0.001 |
| TA06_norank | 0.002 | 0.003 | 0.004 | 0.006 | 0.002 | 0.004 | 0.008 | 0.013 | 0.014 | 0.003 | 0.001 | 0.004 | 0.065 | 0.009 | 0.028 | 0.013 | 0.012 | 0.012 |
| Moraxellaceae_uncultured | 0.063 | 0.087 | 0.015 | 0.000 | 0.006 | 0.000 | 0.001 | 0.000 | 0.000 | 0.007 | 0.004 | 0.006 | 0.009 | 0.000 | 0.000 | 0.000 | 0.000 | 0.000 |
| TRA3-20_norank | 0.013 | 0.010 | 0.008 | 0.002 | 0.006 | 0.003 | 0.028 | 0.005 | 0.016 | 0.011 | 0.010 | 0.014 | 0.002 | 0.004 | 0.023 | 0.002 | 0.012 | 0.016 |
| Kineosporiaceae_unclassified | 0.000 | 0.000 | 0.000 | 0.000 | 0.000 | 0.000 | 0.004 | 0.044 | 0.006 | 0.017 | 0.000 | 0.001 | 0.042 | 0.010 | 0.006 | 0.015 | 0.017 | 0.004 |
| Rhodobacteraceae_unclassified | 0.005 | 0.003 | 0.004 | 0.008 | 0.005 | 0.009 | 0.015 | 0.012 | 0.014 | 0.005 | 0.007 | 0.000 | 0.023 | 0.008 | 0.004 | 0.014 | 0.023 | 0.004 |
| Candidate_division_WS6_norank | 0.001 | 0.003 | 0.002 | 0.001 | 0.002 | 0.002 | 0.010 | 0.014 | 0.008 | 0.021 | 0.012 | 0.011 | 0.003 | 0.003 | 0.010 | 0.009 | 0.014 | 0.017 |
| S0134_terrestrial_group_norank | 0.007 | 0.008 | 0.012 | 0.004 | 0.005 | 0.012 | 0.009 | 0.008 | 0.009 | 0.002 | 0.008 | 0.005 | 0.011 | 0.016 | 0.006 | 0.002 | 0.003 | 0.002 |
| Comamonadaceae_unclassified | 0.004 | 0.007 | 0.009 | 0.004 | 0.005 | 0.014 | 0.030 | 0.008 | 0.010 | 0.002 | 0.007 | 0.000 | 0.010 | 0.005 | 0.004 | 0.002 | 0.001 | 0.002 |
| Acidimicrobiales_uncultured | 0.017 | 0.010 | 0.021 | 0.012 | 0.014 | 0.013 | 0.003 | 0.001 | 0.003 | 0.001 | 0.005 | 0.003 | 0.001 | 0.005 | 0.004 | 0.001 | 0.001 | 0.001 |
| Bacillus | 0.008 | 0.007 | 0.020 | 0.003 | 0.006 | 0.012 | 0.005 | 0.004 | 0.005 | 0.002 | 0.010 | 0.003 | 0.005 | 0.008 | 0.006 | 0.004 | 0.003 | 0.003 |
| Turneriella | 0.001 | 0.000 | 0.001 | 0.000 | 0.000 | 0.000 | 0.000 | 0.000 | 0.009 | 0.024 | 0.000 | 0.045 | 0.007 | 0.000 | 0.000 | 0.002 | 0.017 | 0.006 |
| Pelagibius | 0.009 | 0.005 | 0.011 | 0.017 | 0.016 | 0.014 | 0.008 | 0.001 | 0.010 | 0.001 | 0.003 | 0.005 | 0.001 | 0.004 | 0.004 | 0.001 | 0.000 | 0.002 |
| Parvibaculum | 0.000 | 0.000 | 0.000 | 0.000 | 0.000 | 0.000 | 0.002 | 0.000 | 0.001 | 0.042 | 0.001 | 0.017 | 0.001 | 0.008 | 0.023 | 0.003 | 0.002 | 0.009 |
| AT425-EubC11_terrestrial_group_norank | 0.004 | 0.003 | 0.006 | 0.005 | 0.005 | 0.021 | 0.003 | 0.001 | 0.003 | 0.001 | 0.026 | 0.001 | 0.001 | 0.009 | 0.000 | 0.018 | 0.001 | 0.001 |
| Azotobacter | 0.038 | 0.028 | 0.001 | 0.004 | 0.031 | 0.000 | 0.004 | 0.000 | 0.000 | 0.000 | 0.000 | 0.000 | 0.000 | 0.000 | 0.000 | 0.000 | 0.000 | 0.000 |
| Oceanibaculum | 0.000 | 0.000 | 0.000 | 0.000 | 0.000 | 0.000 | 0.002 | 0.006 | 0.006 | 0.031 | 0.001 | 0.009 | 0.012 | 0.001 | 0.005 | 0.015 | 0.013 | 0.006 |
| Rhizobium | 0.000 | 0.000 | 0.000 | 0.006 | 0.000 | 0.001 | 0.004 | 0.011 | 0.012 | 0.013 | 0.001 | 0.005 | 0.013 | 0.004 | 0.028 | 0.001 | 0.006 | 0.001 |
| Desulfuromonadales_unclassified | 0.014 | 0.003 | 0.009 | 0.002 | 0.006 | 0.009 | 0.008 | 0.004 | 0.005 | 0.006 | 0.011 | 0.009 | 0.002 | 0.001 | 0.009 | 0.001 | 0.003 | 0.003 |
| Hydrogenophaga | 0.002 | 0.001 | 0.000 | 0.000 | 0.001 | 0.000 | 0.005 | 0.005 | 0.005 | 0.015 | 0.019 | 0.002 | 0.025 | 0.001 | 0.000 | 0.005 | 0.007 | 0.003 |
| Hydrogenedentes_norank | 0.003 | 0.003 | 0.002 | 0.002 | 0.003 | 0.003 | 0.004 | 0.003 | 0.002 | 0.005 | 0.003 | 0.004 | 0.009 | 0.004 | 0.002 | 0.018 | 0.021 | 0.004 |
| Sandaracinaceae_uncultured | 0.004 | 0.003 | 0.003 | 0.000 | 0.001 | 0.000 | 0.005 | 0.002 | 0.005 | 0.001 | 0.003 | 0.007 | 0.002 | 0.000 | 0.009 | 0.022 | 0.010 | 0.012 |
| Marmoricola | 0.006 | 0.004 | 0.005 | 0.017 | 0.005 | 0.010 | 0.006 | 0.003 | 0.006 | 0.001 | 0.002 | 0.001 | 0.005 | 0.011 | 0.004 | 0.001 | 0.000 | 0.000 |
| Rhodospirillaceae_unclassified | 0.008 | 0.006 | 0.001 | 0.002 | 0.004 | 0.001 | 0.002 | 0.000 | 0.002 | 0.005 | 0.006 | 0.003 | 0.002 | 0.003 | 0.011 | 0.017 | 0.005 | 0.012 |
| Geoalkalibacter | 0.003 | 0.003 | 0.002 | 0.000 | 0.001 | 0.001 | 0.014 | 0.017 | 0.007 | 0.010 | 0.005 | 0.004 | 0.001 | 0.001 | 0.009 | 0.002 | 0.001 | 0.004 |
| 34P16_norank | 0.014 | 0.012 | 0.005 | 0.012 | 0.010 | 0.002 | 0.000 | 0.000 | 0.001 | 0.000 | 0.001 | 0.002 | 0.000 | 0.005 | 0.006 | 0.000 | 0.000 | 0.014 |
| Opitutus | 0.002 | 0.003 | 0.001 | 0.003 | 0.001 | 0.002 | 0.004 | 0.004 | 0.003 | 0.004 | 0.002 | 0.003 | 0.006 | 0.003 | 0.005 | 0.013 | 0.005 | 0.016 |
| Phenylobacterium | 0.001 | 0.001 | 0.000 | 0.000 | 0.000 | 0.000 | 0.002 | 0.007 | 0.004 | 0.004 | 0.001 | 0.000 | 0.005 | 0.001 | 0.002 | 0.022 | 0.021 | 0.007 |
| Ramlibacter | 0.004 | 0.008 | 0.013 | 0.006 | 0.005 | 0.004 | 0.004 | 0.001 | 0.004 | 0.001 | 0.004 | 0.001 | 0.004 | 0.011 | 0.005 | 0.001 | 0.000 | 0.002 |
| Perlucidibaca | 0.001 | 0.004 | 0.001 | 0.000 | 0.000 | 0.000 | 0.001 | 0.002 | 0.001 | 0.000 | 0.000 | 0.001 | 0.062 | 0.002 | 0.000 | 0.000 | 0.000 | 0.001 |
| Illumatobacter | 0.001 | 0.001 | 0.002 | 0.006 | 0.002 | 0.005 | 0.007 | 0.002 | 0.007 | 0.002 | 0.009 | 0.001 | 0.001 | 0.017 | 0.005 | 0.002 | 0.000 | 0.000 |
| JG30-KF-CM45_norank | 0.003 | 0.001 | 0.002 | 0.004 | 0.001 | 0.006 | 0.005 | 0.003 | 0.004 | 0.002 | 0.004 | 0.002 | 0.006 | 0.017 | 0.004 | 0.003 | 0.002 | 0.001 |
| Achromobacter | 0.000 | 0.000 | 0.000 | 0.000 | 0.000 | 0.000 | 0.001 | 0.008 | 0.009 | 0.012 | 0.001 | 0.007 | 0.005 | 0.003 | 0.004 | 0.002 | 0.006 | 0.013 |
| Nitrosomonadaceae_uncultured | 0.008 | 0.012 | 0.018 | 0.001 | 0.005 | 0.003 | 0.002 | 0.001 | 0.004 | 0.001 | 0.002 | 0.002 | 0.001 | 0.003 | 0.004 | 0.000 | 0.000 | 0.000 |
| Blfdi19_norank | 0.000 | 0.000 | 0.000 | 0.000 | 0.000 | 0.000 | 0.000 | 0.000 | 0.000 | 0.062 | 0.001 | 0.000 | 0.000 | 0.000 | 0.000 | 0.002 | 0.000 | 0.000 |
| Nocardioides | 0.002 | 0.001 | 0.001 | 0.006 | 0.003 | 0.007 | 0.004 | 0.001 | 0.004 | 0.001 | 0.002 | 0.001 | 0.008 | 0.013 | 0.006 | 0.003 | 0.001 | 0.001 |
| Geobacter | 0.013 | 0.001 | 0.003 | 0.003 | 0.014 | 0.009 | 0.000 | 0.000 | 0.000 | 0.001 | 0.005 | 0.000 | 0.000 | 0.000 | 0.000 | 0.000 | 0.011 | 0.000 |
| SubsectionIII_FamilyI_unclassified | 0.002 | 0.003 | 0.000 | 0.000 | 0.000 | 0.002 | 0.001 | 0.000 | 0.000 | 0.001 | 0.031 | 0.001 | 0.002 | 0.011 | 0.000 | 0.006 | 0.000 | 0.001 |
| Sedimentibacter | 0.000 | 0.000 | 0.000 | 0.000 | 0.000 | 0.000 | 0.000 | 0.011 | 0.000 | 0.003 | 0.001 | 0.000 | 0.001 | 0.000 | 0.000 | 0.039 | 0.004 | 0.000 |
| JL-ETNP-Z39_norank | 0.009 | 0.006 | 0.009 | 0.005 | 0.009 | 0.011 | 0.001 | 0.001 | 0.002 | 0.000 | 0.001 | 0.002 | 0.001 | 0.001 | 0.000 | 0.000 | 0.001 | 0.000 |
| MSB-1E8_norank | 0.010 | 0.007 | 0.006 | 0.007 | 0.008 | 0.007 | 0.002 | 0.001 | 0.001 | 0.001 | 0.002 | 0.002 | 0.001 | 0.001 | 0.001 | 0.001 | 0.001 | 0.000 |
| Ardenticatenia_uncultured | 0.004 | 0.005 | 0.003 | 0.002 | 0.004 | 0.004 | 0.004 | 0.001 | 0.004 | 0.004 | 0.013 | 0.006 | 0.000 | 0.001 | 0.001 | 0.000 | 0.001 | 0.000 |
| Tistrella | 0.000 | 0.000 | 0.000 | 0.000 | 0.000 | 0.000 | 0.000 | 0.000 | 0.000 | 0.011 | 0.003 | 0.001 | 0.000 | 0.000 | 0.000 | 0.001 | 0.012 | 0.026 |
| AKYG1722_norank | 0.003 | 0.003 | 0.003 | 0.003 | 0.004 | 0.016 | 0.002 | 0.001 | 0.001 | 0.001 | 0.010 | 0.001 | 0.001 | 0.003 | 0.000 | 0.002 | 0.000 | 0.001 |
| SM2F11_norank | 0.000 | 0.001 | 0.000 | 0.000 | 0.000 | 0.000 | 0.001 | 0.002 | 0.001 | 0.020 | 0.001 | 0.001 | 0.002 | 0.001 | 0.001 | 0.017 | 0.002 | 0.006 |
| Parcubacteria_norank | 0.001 | 0.006 | 0.002 | 0.000 | 0.002 | 0.000 | 0.005 | 0.003 | 0.003 | 0.016 | 0.002 | 0.003 | 0.002 | 0.002 | 0.004 | 0.000 | 0.000 | 0.003 |
| Anaerolinea | 0.001 | 0.002 | 0.002 | 0.001 | 0.001 | 0.002 | 0.001 | 0.016 | 0.006 | 0.001 | 0.001 | 0.006 | 0.009 | 0.001 | 0.000 | 0.003 | 0.002 | 0.001 |
| Pontibacter | 0.001 | 0.003 | 0.005 | 0.010 | 0.003 | 0.027 | 0.000 | 0.000 | 0.000 | 0.001 | 0.002 | 0.000 | 0.001 | 0.001 | 0.000 | 0.000 | 0.000 | 0.000 |
| Arthrobacter | 0.002 | 0.016 | 0.009 | 0.005 | 0.004 | 0.002 | 0.001 | 0.000 | 0.000 | 0.000 | 0.000 | 0.000 | 0.000 | 0.009 | 0.000 | 0.000 | 0.000 | 0.000 |
| Pseudoxanthomonas | 0.000 | 0.000 | 0.000 | 0.004 | 0.001 | 0.001 | 0.001 | 0.003 | 0.013 | 0.003 | 0.001 | 0.003 | 0.001 | 0.005 | 0.005 | 0.001 | 0.004 | 0.000 |
| SM2D12_norank | 0.001 | 0.001 | 0.000 | 0.001 | 0.000 | 0.000 | 0.000 | 0.000 | 0.000 | 0.002 | 0.001 | 0.000 | 0.000 | 0.000 | 0.001 | 0.012 | 0.026 | 0.000 |
| Stella | 0.000 | 0.000 | 0.000 | 0.000 | 0.000 | 0.000 | 0.000 | 0.009 | 0.003 | 0.004 | 0.001 | 0.004 | 0.000 | 0.000 | 0.001 | 0.003 | 0.017 | 0.003 |
| Microbacteriaceae_unclassified | 0.000 | 0.000 | 0.000 | 0.000 | 0.000 | 0.000 | 0.000 | 0.000 | 0.003 | 0.007 | 0.001 | 0.001 | 0.000 | 0.000 | 0.006 | 0.001 | 0.010 | 0.013 |
| Microgenomates_norank | 0.000 | 0.003 | 0.002 | 0.001 | 0.001 | 0.001 | 0.003 | 0.010 | 0.004 | 0.003 | 0.004 | 0.002 | 0.002 | 0.003 | 0.000 | 0.003 | 0.002 | 0.002 |
| Parvularcula | 0.000 | 0.000 | 0.000 | 0.000 | 0.000 | 0.000 | 0.000 | 0.000 | 0.000 | 0.000 | 0.000 | 0.000 | 0.000 | 0.001 | 0.006 | 0.000 | 0.000 | 0.034 |
| Microcoleus | 0.000 | 0.000 | 0.000 | 0.000 | 0.001 | 0.003 | 0.001 | 0.000 | 0.000 | 0.000 | 0.011 | 0.000 | 0.000 | 0.017 | 0.001 | 0.007 | 0.000 | 0.000 |
| Sphingomonadales_unclassified | 0.002 | 0.001 | 0.003 | 0.006 | 0.004 | 0.011 | 0.001 | 0.001 | 0.001 | 0.001 | 0.003 | 0.000 | 0.001 | 0.003 | 0.000 | 0.001 | 0.000 | 0.000 |
| Acholeplasma | 0.000 | 0.000 | 0.000 | 0.000 | 0.000 | 0.000 | 0.000 | 0.002 | 0.000 | 0.000 | 0.000 | 0.000 | 0.002 | 0.000 | 0.000 | 0.007 | 0.024 | 0.004 |
| AKYH478_norank | 0.002 | 0.002 | 0.001 | 0.000 | 0.003 | 0.000 | 0.001 | 0.000 | 0.000 | 0.001 | 0.026 | 0.002 | 0.000 | 0.001 | 0.000 | 0.001 | 0.000 | 0.000 |
| Roseovarius | 0.000 | 0.000 | 0.000 | 0.000 | 0.000 | 0.000 | 0.001 | 0.014 | 0.000 | 0.001 | 0.000 | 0.000 | 0.015 | 0.000 | 0.000 | 0.005 | 0.003 | 0.000 |
| Nitrospira | 0.012 | 0.008 | 0.005 | 0.001 | 0.004 | 0.003 | 0.001 | 0.000 | 0.000 | 0.000 | 0.001 | 0.000 | 0.000 | 0.000 | 0.000 | 0.000 | 0.000 | 0.000 |
| Cytophagaceae_unclassified | 0.000 | 0.000 | 0.000 | 0.000 | 0.001 | 0.000 | 0.000 | 0.000 | 0.000 | 0.001 | 0.000 | 0.019 | 0.000 | 0.005 | 0.001 | 0.002 | 0.001 | 0.006 |
| Myxococcales_unclassified | 0.000 | 0.000 | 0.000 | 0.000 | 0.000 | 0.000 | 0.001 | 0.006 | 0.002 | 0.000 | 0.000 | 0.000 | 0.014 | 0.000 | 0.000 | 0.006 | 0.002 | 0.000 |
| Gemmatimonas | 0.001 | 0.001 | 0.001 | 0.000 | 0.000 | 0.000 | 0.001 | 0.001 | 0.001 | 0.001 | 0.001 | 0.001 | 0.002 | 0.017 | 0.003 | 0.001 | 0.000 | 0.001 |
| Georgenia | 0.001 | 0.000 | 0.000 | 0.001 | 0.001 | 0.001 | 0.002 | 0.001 | 0.000 | 0.000 | 0.001 | 0.000 | 0.011 | 0.005 | 0.000 | 0.003 | 0.002 | 0.000 |
| Phormidium | 0.000 | 0.000 | 0.000 | 0.000 | 0.000 | 0.001 | 0.000 | 0.000 | 0.000 | 0.000 | 0.002 | 0.000 | 0.000 | 0.024 | 0.000 | 0.002 | 0.000 | 0.000 |
| Paenibacillus | 0.001 | 0.020 | 0.001 | 0.001 | 0.001 | 0.000 | 0.001 | 0.000 | 0.000 | 0.000 | 0.000 | 0.000 | 0.000 | 0.000 | 0.000 | 0.001 | 0.000 | 0.000 |
| Porticoccus | 0.000 | 0.000 | 0.000 | 0.000 | 0.000 | 0.000 | 0.000 | 0.000 | 0.000 | 0.018 | 0.000 | 0.001 | 0.000 | 0.000 | 0.000 | 0.001 | 0.000 | 0.005 |
| Draconibacteriaceae_uncultured | 0.000 | 0.000 | 0.000 | 0.000 | 0.000 | 0.000 | 0.000 | 0.001 | 0.000 | 0.001 | 0.000 | 0.000 | 0.002 | 0.000 | 0.000 | 0.011 | 0.005 | 0.000 |
| BSV26_norank | 0.000 | 0.000 | 0.000 | 0.000 | 0.000 | 0.000 | 0.000 | 0.012 | 0.001 | 0.000 | 0.000 | 0.000 | 0.000 | 0.000 | 0.000 | 0.005 | 0.001 | 0.000 |
| Family_XVIII_uncultured | 0.000 | 0.000 | 0.000 | 0.000 | 0.000 | 0.000 | 0.000 | 0.011 | 0.000 | 0.001 | 0.000 | 0.000 | 0.006 | 0.000 | 0.000 | 0.000 | 0.000 | 0.000 |
| Hyphomonas | 0.000 | 0.000 | 0.000 | 0.000 | 0.000 | 0.000 | 0.000 | 0.000 | 0.000 | 0.014 | 0.000 | 0.001 | 0.000 | 0.000 | 0.000 | 0.000 | 0.000 | 0.000 |
| Acidovorax | 0.000 | 0.000 | 0.000 | 0.000 | 0.000 | 0.000 | 0.000 | 0.000 | 0.000 | 0.001 | 0.013 | 0.000 | 0.000 | 0.000 | 0.000 | 0.000 | 0.001 | 0.000 |
| Others | 0.246 | 0.224 | 0.223 | 0.228 | 0.265 | 0.362 | 0.201 | 0.171 | 0.198 | 0.205 | 0.358 | 0.173 | 0.166 | 0.216 | 0.160 | 0.206 | 0.142 | 0.137 |

**Table S3.** Correlation of bacteria and environmental parameters in the low contamination treatment with *S. salsa* (P2C1).

|  | **Anaerolineaceae_uncultured** | **Saccharibacteria_norank** | **Desulfuromonas** | **Mycobacterium** | **Pseudomonas** | **Subgroup_7_norank** | **Blastocatella** | **Azoarcus** | **Subgroup_6_norank** | **Sphingomonas** | **OM1_clade_norank** | **ABS-19_norank** | **Pelagibius** | **AT425-EubC11_terrestrial_group_norank** | **AT425-EubC11** | **Marmoricola** | **AKYG1722_norank** | **Pontibacter** |
| --- | --- | --- | --- | --- | --- | --- | --- | --- | --- | --- | --- | --- | --- | --- | --- | --- | --- | --- |
| SOM | 0.866 | 0.143 | -0.721 | 0.000 | 0.904 | -0.500 | -0.866 | -0.866 | 0.000 | 0.500 | -0.866 | 0.866 | 0.866 | -1.000** | 0.866 | 0.000 | -0.866 | -0.982 |
| TN | -0.945 | -0.842 | 0.996* | -0.756 | -0.269 | 0.982 | 0.189 | 0.945 | 0.756 | -0.982 | 0.945 | -0.189 | -0.945 | 0.655 | -0.189 | -0.756 | 0.945 | 0.786 |
| TP | -0.945 | -0.842 | 0.996* | -0.756 | -0.269 | 0.982 | 0.189 | 0.945 | 0.756 | -0.982 | 0.945 | -0.189 | -0.945 | 0.655 | -0.189 | -0.756 | 0.945 | 0.786 |
| NH_4_-N | 0.866 | 0.143 | -0.721 | 0.000 | 0.904 | -0.500 | -0.866 | -0.866 | 0.000 | 0.500 | -0.866 | 0.866 | 0.866 | -1.000 | 0.866 | 0.000 | -0.866 | -0.982 |
| NO_3_-N | -0.866 | -0.929 | 0.961 | -0.866 | -0.082 | 1.000** | 0.000 | 0.866 | 0.866 | -1.000 | 0.866 | 0.000 | -0.866 | 0.500 | 0.000 | -0.866 | 0.866 | 0.655 |
| pH | 0.945 | 0.842 | -0.996* | 0.756 | 0.269 | -0.982 | -0.189 | -0.945 | -0.756 | 0.982 | -0.945 | 0.189 | 0.945 | -0.655 | 0.189 | 0.756 | -0.945 | -0.786 |
| Crude oil | -0.189 | -0.888 | 0.419 | -0.945 | 0.700 | 0.655 | -0.756 | 0.189 | 0.945 | -0.655 | 0.189 | 0.756 | -0.189 | -0.327 | 0.756 | -0.945 | 0.189 | -0.143 |
| Saturated hydrocarbon | -0.866 | -0.143 | 0.721 | 0.000 | -0.904 | 0.500 | 0.866 | 0.866 | 0.000 | -0.500 | 0.866 | -0.866 | -0.866 | 1.000 | -0.866 | 0.000 | 0.866 | 0.982 |
| Atomatic hydrocarbon | -0.866 | -0.143 | 0.721 | 0.000 | -0.904 | 0.500 | 0.866 | 0.866 | 0.000 | -0.500 | 0.866 | -0.866 | -0.866 | 1.000 | -0.866 | 0.000 | 0.866 | 0.982 |
| asphaltene | 1.000** | 0.619 | -0.971 | 0.500 | 0.569 | -0.866 | -0.500 | -1.000 | -0.500 | 0.866 | -1.000 | 0.500 | 1.000** | -0.866 | 0.500 | 0.500 | -1.000** | -0.945 |

* means significance at p<0.05 level, ** means significance at p<0.01 level.

**Table S4.** Correlation of bacteria and environmental parameters in the medium contamination treatment with *S. salsa* (P2C2).

|  | **Anaerolineaceae_uncultured** | **Saccharibacteria_norank** | **Alcanivorax** | **PYR10d3_norank** | **Pseudomonas** | **C1-B045** | **Azoarcus** | **Proteiniphilum** | **Subgroup_6_norank** | **Sphingomonas** | **Turneriella** | **Parvibaculum** | **Hydrogenophaga** | **Cytophagaceae_unclassified** | **Porticoccus** |
| --- | --- | --- | --- | --- | --- | --- | --- | --- | --- | --- | --- | --- | --- | --- | --- |
| SOM | 0.971 | -0.866 | 0.849 | -0.189 | 0.866 | 0.655 | -0.655 | -0.866 | 1.000 | 0.000 | 0.596 | -0.500 | -0.500 | 0.866 | -0.866 |
| TN | -0.454 | 0.945 | -0.955 | -0.619 | -0.945 | -1.000** | -0.143 | 0.189 | -0.655 | 0.756 | -0.997* | -0.327 | 0.982 | -0.945 | 0.189 |
| TP | -0.454 | 0.945 | -0.955 | -0.619 | -0.945 | -1.000 | -0.143 | 0.189 | -0.655 | 0.756 | -0.997* | -0.327 | 0.982 | -0.945 | 0.189 |
| NH_4_-N | 0.971 | -0.866 | 0.849 | -0.189 | 0.866 | 0.655 | -0.655 | -0.866 | 1.000** | 0.000 | 0.596 | -0.500 | -0.500 | 0.866 | -0.866 |
| NO_3_-N | 0.971 | -0.866 | 0.849 | -0.189 | 0.866 | 0.655 | -0.655 | -0.866 | 1.000** | 0.000 | 0.596 | -0.500 | -0.500 | 0.866 | -0.866 |
| pH | -0.971 | 0.866 | -0.849 | 0.189 | -0.866 | -0.655 | 0.655 | 0.866 | -1.000** | 0.000 | -0.596 | 0.500 | 0.500 | -0.866 | 0.866 |
| Crude oil | 0.817 | -0.189 | 0.156 | -0.866 | 0.189 | -0.143 | -1.000** | -0.945 | 0.655 | 0.756 | -0.217 | -0.982 | 0.327 | 0.189 | -0.945 |
| Saturated hydrocarbon | 0.000 | 0.693 | -0.717 | -0.908 | -0.693 | -0.891 | -0.577 | -0.277 | -0.240 | 0.971 | -0.923 | -0.721 | 0.961 | -0.693 | -0.277 |
| Atomatic hydrocarbon | 0.961 | -0.500 | 0.470 | -0.655 | 0.500 | 0.189 | -0.945 | -1.000** | 0.866 | 0.500 | 0.115 | -0.866 | 0.000 | 0.500 | -1.000** |
| asphaltene | -0.961 | 0.500 | -0.470 | 0.655 | -0.500 | -0.189 | 0.945 | 1.000** | -0.866 | -0.500 | -0.115 | 0.866 | 0.000 | -0.500 | 1.000** |
| colloid | -0.999* | 0.756 | -0.733 | 0.371 | -0.756 | -0.500 | 0.786 | 0.945 | -0.982 | -0.189 | -0.434 | 0.655 | 0.327 | -0.756 | 0.945 |

* means significance at p<0.05 level, ** means significance at p<0.01 level.

**Table S5.** Correlation of bacteria and environmental parameters in the high contamination treatment with *S. salsa* (P2C3).

|  | Desulfuromonas | Alcanivorax | Cytophagaceae_uncultured | Azoarcus | TRA3-20_norank | Turneriella | Sandaracinaceae_uncultured | Rhodospirillaceae_unclassified | Opitutus | Tistrella | SM2F11_norank | SM2D12_norank | Parvularcula |
| --- | --- | --- | --- | --- | --- | --- | --- | --- | --- | --- | --- | --- | --- |
| SOM | 0.500 | 0.655 | 0.893 | -0.786 | 0.982 | 0.327 | -0.756 | -0.756 | 0.655 | 0.867 | -0.327 | -0.500 | 0.945 |
| TN | 0.945 | 0.866 | 0.610 | -0.756 | 0.000 | -0.866 | 0.500 | 0.500 | 0.866 | 0.189 | 0.866 | -0.945 | 0.500 |
| TP | -0.945 | -0.866 | -0.610 | 0.756 | 0.000 | 0.866 | -0.500 | -0.500 | -0.866 | -0.189 | -0.866 | 0.945 | -0.500 |
| NH_4_-N | -0.786 | -0.655 | -0.317 | 0.500 | 0.327 | 0.982 | -0.756 | -0.756 | -0.655 | 0.143 | -0.982 | 0.786 | -0.189 |
| NO_3_-N | -0.929 | -0.982 | -0.980 | 1.000** | -0.655 | 0.327 | 0.189 | 0.189 | -0.982 | -0.786 | -0.327 | 0.929 | -0.945 |
| pH | 0.500 | 0.655 | 0.893 | -0.786 | 0.982 | 0.327 | -0.756 | -0.756 | 0.655 | 0.787 | -0.327 | -0.500 | 0.945 |
| Crude oil | 0.189 | 0.000 | -0.381 | 0.189 | -0.866 | -0.866 | 0.956 | 0.957 | 0.000 | -0.756 | 0.866 | -0.189 | -0.500 |
| Saturated hydrocarbon | -1.000** | -0.982 | -0.836 | 0.929 | -0.327 | 0.656 | -0.189 | -0.189 | -0.982 | -0.500 | -0.656 | 0.756 | -0.756 |
| Atomatic hydrocarbon | 0.945 | 0.866 | 0.610 | -0.756 | 0.000 | -0.866 | 0.500 | 0.500 | 0.866 | 0.189 | 0.866 | -0.945 | 0.500 |
| asphaltene | -0.891 | -0.961 | -0.994 | 0.996 | -0.721 | 0.240 | 0.277 | 0.277 | -0.961 | -0.839 | -0.240 | 0.891 | -0.971 |

* means significance at p < 0.05 level, ** means significance at p < 0.01 level.
